# Selective Effects of mTOR Inhibitor Sirolimus on Naïve and CMV-Specific T Cells Extending Its Applicable Range Beyond Immunosuppression

**DOI:** 10.3389/fimmu.2018.02953

**Published:** 2018-12-17

**Authors:** Szilvia Bak, Sabine Tischer, Anna Dragon, Sarina Ravens, Lars Pape, Christian Koenecke, Mathias Oelke, Rainer Blasczyk, Britta Maecker-Kolhoff, Britta Eiz-Vesper

**Affiliations:** ^1^Hannover Medical School, Institute for Transfusion Medicine, Hannover, Germany; ^2^Hannover Medical School, Institute of Immunology, Hannover, Germany; ^3^Department of Pediatric Nephrology, Hannover Medical School, Hannover, Germany; ^4^Department of Hematology, Hemostasis, Oncology and Stem Cell Transplantation, Hannover Medical School, Hannover, Germany; ^5^Department of Pathology, John Hopkins School of Medicine, Baltimore, MD, United States; ^6^NexImmune Inc., Gaithersburg, MD, United States; ^7^Department of Pediatric Hematology and Oncology, Hannover Medical School, Hannover, Germany

**Keywords:** HCMV, antiviral T cells, mTOR inhibitor, personalized immunosuppression, transplantation, sirolimus

## Abstract

Cytomegalovirus (CMV) infection/reactivation remains among the most important complications of immunosuppression after transplantation. However, recent clinical observations indicate that mammalian target of rapamycin (mTOR) inhibition with sirolimus may improve the outcome of CMV complications. Underlying mechanisms of this observation, particularly the effect of sirolimus on naïve- and CMV-specific cytotoxic CD8^+^ T-cell (CMV-CTL) functionality is still undiscovered. Here, the influence of sirolimus on naïve and memory CMV-CTLs was determined by CD3/CD28 crosslinking and alloreactivity assays. After stimulating CMV-CTL with HLA-A^*^02:01-restricted CMVpp65-peptide loaded artificial antigen-presenting cells (aAPCs), we measured the effect of sirolimus on T-cell proliferation, phenotype, and functionality. Sirolimus significantly improved CMV-specific effector memory T-cell function and negatively influenced naïve T cells. This unique mechanism of action was further characterized by increased secretion of interferon-gamma (IFN-γ), granzyme B (GzB) and enhanced target-cell-dependent cytotoxic capacity of activated CMV-CTLs. Next-generation-sequencing (NGS) was applied to monitor T-cell receptor (TCR)-repertoire dynamics and to verify, that the increased functionality was not related to sirolimus-resistant CTL-clones. Instead, modulation of environmental cues during CMV-CTL development via IL-2 receptor (IL-2R)-driven signal transducer and activator of transcription-5 (STAT-5) signaling under mTOR inhibition allowed fine-tuning of T-cell programming for enhanced antiviral response with stable TCR-repertoire dynamics. We show for the first time that sirolimus acts selectively on human naïve and memory T cells and improves CMV-specific T-cell function via modulation of the environmental milieu. The data emphasize the importance to extend immune monitoring including cytokine levels and T-cell functionality which will help to identify patients who may benefit from individually tailored immunosuppression.

## Introduction

Immunosuppressive therapy to deplete T cells, redirect T-cell trafficking, or terminate T-cell response pathways after solid organ (SOT) and hematopoietic stem cell transplantation (HSCT) is mainly used to prevent graft rejection or severe graft-vs.-host disease (GvHD) (1–3). Immunocompromised patients are highly susceptible to viral infection and reactivation by endogenous herpes viruses such as cytomegalovirus (CMV) and Epstein-Barr virus (EBV), which are associated with high morbidity and mortality (4, 5). Current treatment strategies involve administering effective antiviral drug therapies, reducing the degree of immunosuppression, or changing the individual immunosuppressive drug regimen in order to restore virus-specific T cell-mediated immune responses (4, 6–10).

The immunosuppressive drug sirolimus was first discovered as an antifungal metabolite in *Streptomyces hygroscopicus* in 1975 (11), and was later found to potently inhibit the proliferation of immune cells such as T cells and dendritic cells (DCs) (12). Its target is the cellular kinase called mammalian target of rapamycin (mTOR), which is present in two functionally district complexes: complex 1 (mTORC1, sirolimus-sensitive) and complex 2 (mTORC2). Similar to other mTOR inhibitors (so-called rapalogs) such as everolimus, sirolimus prevents the translation of proteins that promote cell survival and proliferation by engaging with FK506-binding protein (FKBP). The sirolimus-FKBP complex binds to the sirolimus-sensitive mTORC1-protein complex and thus inhibits downstream phosphorylation activities, resulting in the blockade of G1/S cell cycle progression (13–17). The drug further mediates immunosuppressive function by attenuating signaling through the interleukin-2 receptor (IL-2R) and other cytokine receptors (12).

In 2005, Ozaki et al. were the first to report that sirolimus monotherapy results in better outcomes in renal transplant patients with CMV disease than standard calcineurin inhibitor-based immunosuppression (18). This observation was strengthened by accumulating evidence of better control of CMV viremia in sirolimus-treated patients following HSCT and SOT (18–22). Initially, it was speculated that by targeting the mTOR complex during the lytic phase of CMV infection, sirolimus abrogates the infection, and inhibits reactivation since CMV utilizes the mTORC1 pathway for viral replication (18). However, recent studies have shown that the favorable outcomes after transplantation are not associated with the direct molecular blockade of CMV reactivation, but can be attributed to indirect effects on the immune system (19). In 2009, two independent groups reported that sirolimus exerts dose-dependent immunostimulatory effects on CD8^+^ memory T cells in mice and rhesus macaques exposed to viral pathogens (12, 23, 24). High-dose sirolimus suppressed CD8^+^ T-cell expansion, whereas the quality and quantity of T-cell response was dependent on the duration and timing of treatment. When studying the immunostimulatory effects of sirolimus on bacterial-induced CD8^+^ T-cell responses against skin transplants in a transgenic mouse system, Ferrer et al. (25) observed that sirolimus boosted antigen-specific T-cell responses to the pathogen, but not to the transplant. These effects seem to be intrinsic to T cells and influenced by the environment in which the antigen is presented.

Further studies demonstrated the link between the unique metabolic requirements of T cells and the ability of mTORC1 to integrate environmental cues involved in direct T-cell differentiation and function during sirolimus treatment (26–28). These results indicate that the drug functions as a signaling component downstream of T-cell receptor (TCR)/CD3-mediated activation. In addition to TCR-stimulation, co-stimulation, and IL-2R signaling also appear to play an important role in the effects of sirolimus on T-cell functionality (26, 29). Despite sirolimus-sensitive mTORC1, IL-2 signaling in T cells is also mediated by the signal transducer and activator of transcription 5 (STAT-5) (30–32). Although many reports focus on the role of mTORC1 signaling, cross-talk between these key regulators and the signal that drives T-cell function in the presence of sirolimus have not been defined yet.

In this study, diligent characterization of the effects mediated by sirolimus and its interactions with TCR, IL-2R, mTORC1, and STAT-5 on the functionality of CMV-specific CD8^+^ cytotoxic T lymphocytes (CTLs) and naïve T cells was assessed. To exclude the influence of sirolimus on other cells besides T cells, artificial antigen-presenting cells (aAPCs) loaded with HLA-A^*^02:01-restricted CMVpp65 peptide (A02pp65_p_) was used (33, 34).

We found that naïve T cells showed no significant response to treatment with sirolimus. In contrast on memory T cells sirolimus had differential effects on key elements of T-cell activation and function such as (1) the dynamics of the TCR repertoire, (2) the phosphorylation of proteins involved in TCR/mTORC1/IL-2R signaling, and (3) the expression of micro-RNAs (miRNAs, e.g., miRNA-21) and effector genes like granzyme B (GzB) and interferon-gamma (IFN-γ). The modulation of environmental cues during antiviral memory T-cell development through the activation of IL-2R driven STAT-5 signaling under the cover of mTORC1 inhibition allows the fine-tuning of antiviral T-cell programming for improved CMV-specific T-cell response.

These results suggest a need to optimize the monitoring of immunosuppressed patients with an elevated risk of pathogen infection or reactivation by determining serum IL-2 or IL-2R subunit-sharing cytokine levels and antigen-specific T-cell functionality for further individualization of immunosuppressive therapy.

## Materials and Methods

### Isolation of PBMCs and T Cells

Experiments were performed with residual blood samples from platelet (PLT) apheresis disposables used for routine PLT collection of regular anonymous healthy donors of the Hannover Medical School (MHH) Institute for Transfusion Medicine. Informed consent was obtained from all donors following approval by the Ethics Committee of MHH (ethical number: 3639-2017, 2744-2015), and trial subject data were treated as confidential information protected by medical confidentiality. Peripheral blood mononuclear cells (PBMCs) were isolated from HLA-A^*^02:01-positive CMV-seropositive donors by discontinuous-gradient centrifugation. Untouched CD3^+^ and CD8^+^ T cells were enriched by magnetic cell sorting (MACS) using negative selection kits (Miltenyi Biotec, Bergisch, Gladbach, Germany), according to the manufacturer‘s instructions. The purity was routinely higher than 90%, as determined by flow cytometry. Magnetically labeled non-T cells were collected from CD3^+^ T-cell isolation and were used as target cell population in alloreactivity approach.

### Alloreactivity and CD3/CD28 Crosslinking Approach

To investigate the effects of sirolimus on human naïve and memory CD8^+^ T-cell populations, alloreactivity assay was performed using 1 × 10^5^ CD3^+^ T cells stimulated for 2 days with 5 × 10^5^ allogeneic CD3^−^ cells which were simultaneously collected during CD3^+^ T-cell isolation and then irradiated by high dose gamma irradiation (30Gy). For antigen-independent stimulation 5 × 10^5^ isolated CD8^+^ T cells were stimulated on anti-CD28-coated (CD28.2, Becton Dickinson (BD, Heidelberg, Germany) 48-well plates or with human T activator CD3/CD28 Dynabeads (Thermo Fisher Scientific, Waltham, MA) for 3 days according to the manufacturer's instructions. Cell culture media (RPMI 1640, Lonza, Basel, Switzerland) was supplemented with 10% heat-inactivated human AB serum (c.c.pro Oberdorla, Germany), IL-2 (50 U/ml, PeproTech, Hamburg, Germany) in the presence or absence of sirolimus (Sigma-Aldrich by Merck, Darmstadt, Germany) at the recommended therapeutic concentration (10 ng/ml) (13, 35). T cells were analyzed for their phenotype and expression of activation markers by flow cytometry.

### Generation of CMV-Specific CD8^+^ T Cells Using aAPC Beads

To examine the direct effects of sirolimus on CMV-specific CTLs, aAPC beads were used. These aAPCs were generated by coupling HLA-A^*^02:01 molecules (DimerX, BD), loaded with anti-CD28 mAbs (BD) and HLA^*^02:01-restricted CMVpp65_495−503_ peptide (NLVPMVATV, A02pp65_p_, ProImmune, Oxford, UK) onto M-450 Epoxy beads (Thermo Fisher Scientific, Waltham, MA, USA), as previously described (33). The beads were ready to use and were stored at 4°C up to 6 months. Isolated CD8^+^ T cells were cultured at the recommended 1 to 1 cells to aAPC beads density for 7 days in aAPC medium [(RPMI 1640 (Lonza) supplemented with 1% sodium pyruvate (c.c.pro), 5 or 10% heat-inactivated human AB serum (c.c.pro), 0.4% MEM vitamins and 1% non-essential amino acids (Thermo Fisher Scientific)] in the presence or absence of sirolimus (0.5–1000 ng/ml, Sigma-Aldrich by Merck). Sirolimus and aAPCs were added at the same time to the isolated CD8^+^ T cells on day 0. The aAPC medium was supplemented with IL-2 (50 U/ml) on days 0 and 3 for the generation of CMV-specific CD8^+^ T cells. IL-7/IL-12/IL-15 or IL-21 cytokines (each 10 ng/ml; all PeproTech) were added independently as indicated in the result section and were used to replace IL-2 for the evaluation of IL-2 and sirolimus-related expansion and functionality of CMV-specific T cells. The frequency of A02pp65_p_-positive (CMV-specific multimer^+^) CD8^+^ T cells was assessed on day 7 using peptide major histocompatibility complex (pMHC) multimer staining and further analyses were performed as described in detail below.

### Flow Cytometry Analysis, Multimer Staining, and Phenotyping

Phenotypic characterization of T cells was carried out after alloreactivity, CD3/CD28 crosslinking approaches and aAPC stimulation, using the following antibodies: anti-CD3-peridinin-chlorophyll (PerCp) (SK7), anti-CD8-AlexaFluor700 (AF-700) (SK1), anti-CD25- allophycocyanin(APC)/phycoerythrin (PE)/Cy7/BV421 (BC96), anti-CD45RA-PE/Cy7/BV510 (HI100), anti-CD62L-APC/Cy7 (DREG-56), anti-CD69-APC/Cy7/PE/Cy7 (FN50), anti-137-APC (4B4-1), anti-CD366-APC/Cy7 (F38-2E2), anti-CD223-fluorescein-isothiocyanate (FITC) (11C3C65), anti-CD152-PE/Cy7 (L3D10) (all BioLegend, San Diego, CA, USA) and anti-CD279-PE (EH12.1), anti-CD154-PE (TRAP1) (BD). All flow cytometry analyses were performed using the FACS Canto 6c and 10c systems (BD) and BD FACSDiva Software version 8.0.1. At least 10,000 events were acquired in the CD3^+^ or CD8^+^ gate. Gates were set based on the light-scatter properties of lymphocytes.

Multimer staining was performed to monitor the frequency of A02pp65_p_-positive (CMV-specific) CD8^+^ T cells. It was assessed before and after aAPC stimulation using PE or APC-conjugated HLA-A^*^02:01/CMVpp65_p_-specific (Immudex, Copenhagen, Denmark) dextramers. To be considered positive (multimer^+^), the sample had to (1) be a well-defined cell population and/or (2) contain ≥0.5% multimer^+^CD8^+^ T cells.

### Cell Counting, T-Cell Proliferation, and Cell Death

Trypan blue dye (Thermo Fisher Scientific) exclusion technique for counting of living cells manually was performed using bifocal light microscope before and after T-cell stimulation assays. Cell proliferation was monitored by carboxyfluorescein succinimidyl ester (CFSE) labeling (Thermo Fisher Scientific) at final concentration 1 μM on day 0 and CFSE-labeled cells were stimulated 7 days according to aAPC approach. Dead cells were excluded by 7-amino-actinomycin (7AAD) (BioLegend) staining in combination with the following cell surface antibodies: anti-CD8-PE/Cy7 (SK1) (BioLegend), anti-CD3-APC (SK7) (BD). CFSE dilution and 7AAD were analyzed by flow cytometry.

### Intracellular Cytokine Staining

After 7 days of aAPC stimulation and sirolimus treatment, cells were re-stimulated with 10 μg/ml A02pp65_p_ at a density of 1–2 × 10^5^ cell/well for 1 h at 37°C and incubated with Brefeldin A (1:1,000, BioLegend) for additional 4 h at 37°C. Expression of intracellular cytokines were assessed by pMHC multimer and surface staining for CD3 and CD8 following intracellular staining with anti-Granzyme B-PacificBlue (GB11), anti-TNFα-PE/Cy7 (Mab11) (BioLegend), anti-IFNγ-PE (45.15) (Beckmann Coulter, Brea, CA, USA) using IntraPrep Kit (Beckmann Coulter) according to the manufacturer‘s instructions. Briefly, following 5 h peptide re-stimulation, multimer, and surface antibody staining were performed and then the cells were permeabilized by addition of IntraPrep Reagent 1 and 2, subsequently. Cells were washed afterwards, stained with intracellular antibodies, and analyzed by flow cytometry.

### IFN-γ ELISpot Assay

Antigen-specific IFN-γ-producing CD8^+^ T cells were determined after 7 days of aAPC stimulation and treatment by IFN-γ Enzyme Linked Immuno Spot Assay (ELISpot) as previously described (36), using pre-coated IFN-γ EliSpot plates (Lophius Biosciences, Regensburg, Germany). Briefly, 2.5 × 10^3^ CD8^+^ T cells were plated in 125 μl aAPC media/well and incubated overnight with 10 μg/ml A02pp65_p_ or left unstimulated (negative control). Spots were developed based on the manufacturer's recommendation and data were acquired on an “AID iSpot Reader System” with “AID EliSpot Software Version 7.0” and spot counting was performed with “AID EliSpot Software Version 8.0.” All spot counts are mean values from duplicates and expressed as spot-forming unit (SFU) or SFU per 1,000 multimer^+^CD8^+^ T cells, respectively.

### Multiplex Cytokine Profiling

The secretion levels of effector molecules in the T-cell supernatants after culture of 7 days aAPC stimulation with or without sirolimus treatment in the presence of IL-2 were determined by LEGENDplex™ bead-based immunoassay (BioLegend) following overnight A02pp65_p_ re-stimulation. The LEGENDplex™ Human CD8/NK Panel was used to quantify simultaneously 13 human cytokines, including IL-2, IL-4, IL-10, IL-6, IL-17A, tumor necrosis factor alpha (TNF-α), soluble Fas (sFas), sFas ligand (sFasL), IFN-γ, granzyme A (GzA), GzB, perforin, and granulosyn according to manufacturer's protocol.

### CD107a Degranulation Assay

Cytotoxicity of CMV-specific CD8^+^ T cells was assessed by detecting cell surface expression of CD107a. On day 7, 2.5 × 10^5^ aAPC stimulated and sirolimus treated cells (as previously described) were re-stimulated with A02pp65_p_. Anti-CD107a-PE/Cy7 (H4A3) and Monensin (1:1,000, both from BioLegend) were added and cells were incubated for 4 h at 37°C before cell surface staining with anti-CD3-FITC (UCHT1) and anti-CD8-APC (SK1) (both BioLegend) was performed. pMHC multimer staining was assessed before re-stimulation for 10 min at 37°C. Data were acquired by flow cytometry.

### Evaluation of Cytotoxicity in Response to Target Cell Recognition

Cytotoxicity of the 7 day aAPC stimulated and sirolimus treated CMV-specific CD8^+^ effector T cells was evaluated in the presence of HLA-A^*^02:01 transduced and CFSE (final concentration 1 μM) labeled K562 target cells (1.5 × 10^7^ cells) For peptide-loading, target cells were re-suspended in aAPC media and plated into 24-well plates at a cell density of 2.5 × 10^6^ cells/well. Peptide (A02pp65) was added at a concentration of 10 μg/ml and incubated overnight at 37°C. On day 7, following aAPC stimulation and sirolimus treatment in the presence of IL-2 or IL-15, CMV-specific, effector and peptide loaded K562 target cells were cocultured for 5 h at 37°C in 96 well-plates in fresh aAPC media containing IL-2 or IL-15, respectively. Effector to target (E:T) ratios of 1:1, 5:1, and 10:1 were obtained by setting target cell number constant (2.5 × 10^4^ cells/well). Specific lysis of target cells was detected by 7AAD staining and data were acquired using flow cytometry.

### Phosphorylation Analysis

Following 7 days of aAPC stimulation and sirolimus treatment CD8^+^ T cells were re-stimulated with 10 μg/ml peptide (A02pp65) for 1 h and phosphorylation of extracellular regulated kinase 1/2 (pERK1/2), protein kinase B on Ser473 (pAkt^Ser473^), on Thr308 (pAkt^Thr308^), ribosomal protein 6 (pS6), STAT-5 (pSTAT-5) was evaluated by phospho-flow cytometry. Phosphorylation was determined by surface staining with anti-CD3-FITC (UCHT1) (BioLegend), anti-CD8-PerCP/Cy5.5 (RPA-T8) (BD), followed by fixation (Fix Buffer I, BD), permeabilization with Perm Buffer III (BD), and intracellular staining with anti-pS6-AlexaFlour647 (N7-548), anti-pERK1/2-AlexaFlour647 (20A), anti-pSTAT5-AlexaFlour647 (47/STAT5), or immunoglobulin (Ig)G1 AlexFlour647 isotype control (MOPC-21), anti-pAkt(T308)-PE (J1-223.371), anti-pAkt(S473)-PE (M89-61), or IgG1 PE isotype control (MOPC-21) (all BD).

### STAT-5 Inhibition

To investigate the mode of action of STAT-5, CMV-specific CD8^+^ T cells were first stimulated for 7 days with aAPCs and treated with or without sirolimus in the presence of IL-2 or IL-15. Stimulated CD8^+^ T cells were incubated with STAT-5 inhibitor (STAT-5i, Merck Millipore) at a concentration of 50 μg/ml for ~20 h at 37°C. In addition to intracellular cytokine staining (ICS), T-cell STAT-5 and S6 phosphorylation was determined by phospho-flow cytometry and fluorescence microscopy as described below.

### Immunofluorescence Microscopy

A total of 1.5 × 10^5^ CD8^+^ T cells were re-stimulated with A02pp65_p_ for 1 h or left unstimulated. Thereafter, cells were stained with PE-conjugated pMHC multimers for 30 min at RT. Each sample was fixed then with Fix Buffer I and permeabilized with Perm Buffer III (both from BD). Cells were stained with anti-pSTAT5/b (5G4) primary antibody (at a final concentration of 1 μg/ml) and IgG-FITC secondary antibody (both from Santa Cruz Biotechnology, Dallas, TX, USA) followed by staining with anti-pS6-AlexaFlour647 (N7-548) (BioLegend). Staining was performed for 30 min at 4°C. Between each individual steps, cells were subsequently washed twice with PBS^+^1%BSA. Cells were placed onto microscope slides and after mounting of the coverslip with mounting medium (Dianova, Hamburg, Germany), samples were analyzed by using Olympus IX81 fluorescent microscope (Olympus, Shinjuku, Tokyo, Japan) at magnification 60 ×.

### Gene and miRNA Expression Analysis

Total RNA from CD8^+^ T cells after aAPC stimulation and treatment (as previously described: after re-stimulation with 10 μg/ml CMVpp65_p_ for overnight) was isolated using mirVana RNA isolation Kit (Thermo Fisher Scientific). cDNA was reverse-transcribed by either the microRNA Transcriptions Kit or the High-capacity cDNA Reverse Transcription Kit (Thermo Fisher Scientific), according to the manufacturer‘s instructions. Expression of miR-21, miR-155, miR-181a, perforin, cyclin D1 (Bcl-1), suppressor of cytokine signaling 1 (SOCS1), phosphatidylinositol-3,4,5-trisphosphate 5-phosphatase-1 (SHIP-1), T-cell associated transcription factor (T-bet), mitogen activated protein kinase 1 (MAPK1)/ERK, eomesodermin (EOMES), and Ki-67 were quantified by inventoried mixes (Thermo Fisher Scientific). Glyceraldehyde-3-phosphate dehydrogenase (GAPDH) and miR-191 served as internal control.

Global gene expression analysis was performed on multimer-sorted CMV-specific CD8^+^ T cells (expanded and treated with sirolimus for 7 days in the presence of IL-2 as described above) following overnight aAPC re-stimulation. CMV-specific CD8^+^ cells were sorted based on their multimer-specificity by high speed flow cytometry sorters at the Research Facility Cell Sorting of MHH. The Microarray utilized in this study represents a refined version of the Whole Human Genome Oligo Microarray 4 × 44K v2 (Design ID 026652, Agilent Technologies), called “026652QM_RCUG_HomoSapiens” (Design ID 084555) developed by the Research Core Unit Genomics (RCUG) of MHH. Microarray design was created at Agilent's eArray portal using a 1 × 1 M design format for mRNA expression as template. All non-control probes of design ID 026655 have been printed four times within a region comprising a total of 181560 Features (170 columns × 1,068 rows). Four of such regions were placed within one 1M region giving rise to four microarray fields per slide to be hybridized individually (Customer Specified Feature Layout). Control probes required for proper Feature Extraction software operation were determined and placed automatically by eArray using recommended default settings. Measurements of on-chip replicates were averaged and normalized by quantile normalization approach. Then clustering and heat map were created using the Morpheus web-based tool. GeneCards® database was used to receive genomic and proteomic information about the particular genes.

### TCR Sequencing

For mRNA-isolation of flow cytometry sorted cells the Qiagen Micro Kit was used, following rapid amplification of cDNA ends using the Smarter 5'RACE cDNA amplification kit (Clontech 634923) according to the recommended protocol. Per sample 5 μl RNA was used for cDNA synthesis. Next, complementarity-determining region 3 (CDR3) regions of the human TCR beta chain were amplified through gene-specific primers for the constant region of the beta (β)-chain (GCACACCAGTGTGGCCTTTTGGG) and the introduced SMARTER oligonucleotide (CTAATACGACTCACTATAGGGC) using the Advantage 2 PCR kit (Clontech 639206) in a 50 μl reaction. Primer sequences contain 16 S Illumina overhang adapter sequences. Cycling conditions were as following: 120 s 95°C; 30 times 30 s 95°C, 45 s 64°C, 60 s 72°C; 60 s 72°C. Generated PCR amplicons were agarose gel purified. Next, samples were labeled with Nextera Illumina Index reads within 10 additional PCR cycles using the Advantage 2 PCR kit (CLontech) and purified with Agencourt AMPpure XP beads (Beckman Coulter) and. subjected to Illumina MiSeq analysis using V2 500 cycles or V3 600 cycle paired-end sequencing reagent. Obtained Fastq files were annotated according to IMGTHighV/quest. For downstream bioinformatics analysis only productive reads were taken into consideration. Individual CDR3 sequences were ranked according to their abundance within the respective samples. For multisample comparison obtained reads of CDR3 sequences were normalized to all productive reads per sample. Shannon diversity indices were calculated using the R library “vegan” prior to normalization to 5,000 productive sequences.

### Patients and Treatment Regimen

PBMCs from *in vivo* sirolimus-treated kidney- (*n* = 3) and stem cell-transplanted (*n* = 2) patients or from healthy individuals (*n* = 6) with or without sirolimus treatment (10 ng/ml) were rested overnight at 37°C. Patient information is summarized in Table S1. Thereafter, analysis of STAT-5 and S6 phosphorylation were assessed as described previously following 15 min CD3/CD28 Dynabeads stimulation in the presence or absence of IL-2. Additionally, intracellular cytokine staining was performed following 5 h incubation/stimulation under the aforementioned conditions. Phosphorylation of S6 and STAT-5 and cytokine expression was assessed as described above on gated CD8^+^ T cells by flow cytometry.

### Statistics

Statistical analyses were performed in GraphPad Prism version 7.0 software (GraphPad Software, San Diego, CA, USA) using two-paired Student's *t*-test or two-way analysis of variance. Levels of significance were expressed as *p*-values [^*^p < 0.05, ^**^p < 0.01, ^***^p < 0.001, ^****^p < 0.0001, not significant (n.s.)].

## Results

### Expression of Activation Marker on Sirolimus-Treated Memory T Cells Is Induced by TCR Activation and IL-2 Supplementation

**Figure 1:**
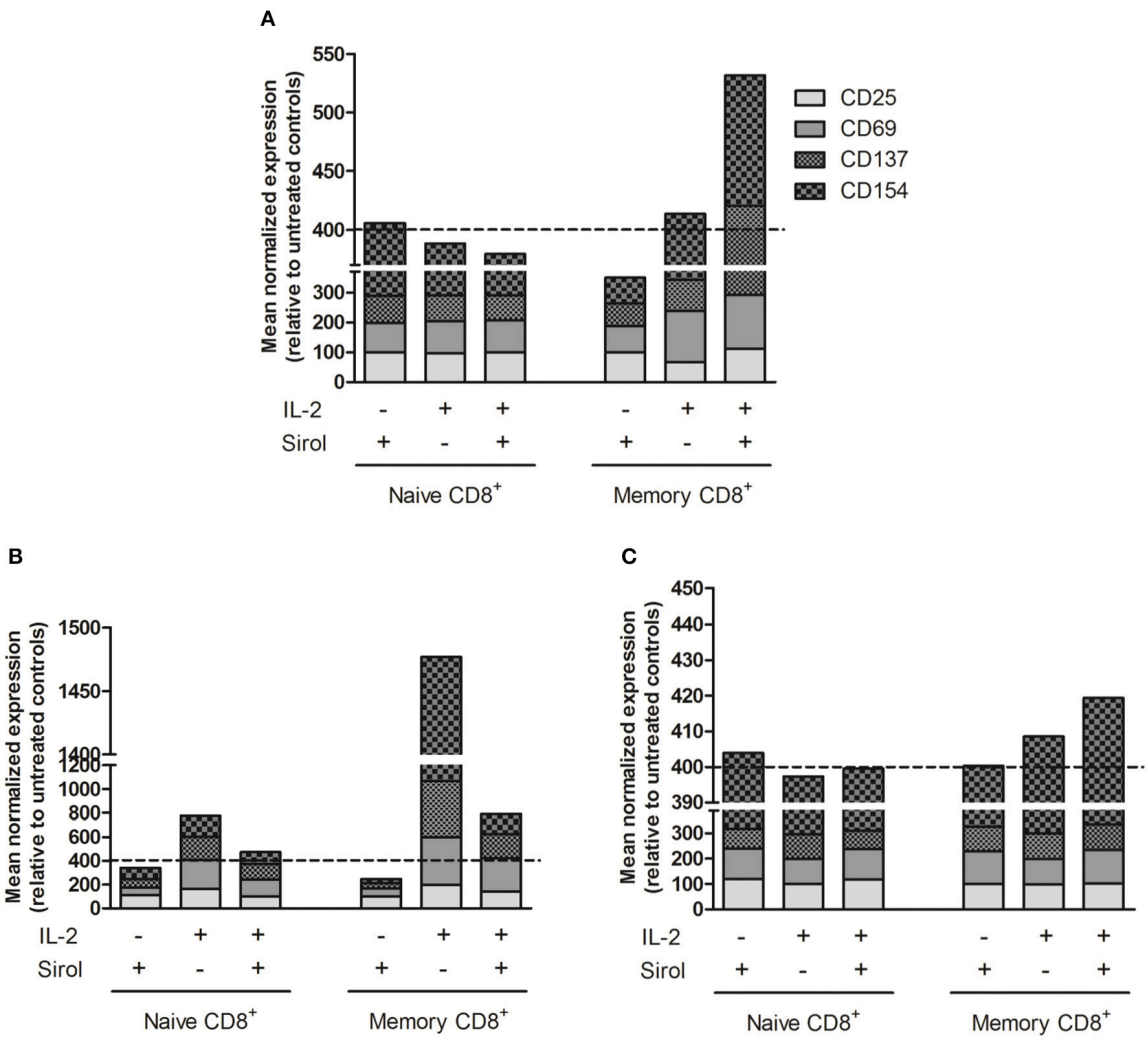
Activation marker expression is induced on sirolimus-treated memory T cells after TCR activation and IL-2 supplementation. Surface activation marker expression was measured on naïve CD8^+^CD45RA^+^CD62L^+^ and memory CD8^+^CD45RA^−^ T cells after treatment with (+) or without (–) sirolimus (Sirol, 10 ng/ml) in the presence (+) or absence (–) of IL-2 (50 U/ml). Shown are mean normalized percentages of activation markers relative to untreated controls (indicated as dashed lines). **(A)** Stimulation on allogeneic irradiated CD3^−^ cells (*n* = 6). **(B)** Stimulation on anti-CD28-coated plates (*n* = 5). **(C)** Stimulation with anti-CD3/CD28 beads (*n* = 3).

As the purpose of immunosuppression is to prevent complications like graft rejection and GvHD, which are mainly caused by alloreactive naïve T cells, the selective effect of sirolimus was investigated on human naïve and memory CD8^+^ T-cell populations. T-cell activation marker expression was analyzed with an alloreactivity assay using CD3^+^ T cells stimulated with irradiated allogeneic CD3^−^ cells (Figure 1A) after co-stimulation with anti-CD28-coated plates (Figure 1B) and stimulation by anti-CD3/CD28 cross-linking (Figure 1C) in the presence of IL-2 and sirolimus at the recommended therapeutic concentration (10 ng/ml). On naïve CD8^+^CD45RA^+^CD62L^+^ T cells, the mean normalized percentage of cells (sum) expressing CD25, CD69, CD137, and CD154 relative to that in untreated controls (equalized to a sum of 400%) was not affected by sirolimus alone (405.6%) and was moderately downregulated by supplemental IL-2 alone (388.1%) and by sirolimus and IL-2 combined (379.4%) (Figure 1A). Sirolimus alone led to a decrease in memory CD8^+^CD45RA^−^ T-cell activation (349.8%). Interestingly, this suppressive effect was overcome by combining sirolimus with IL-2 (531.8%). These results imply that the immunosuppressive effects of sirolimus on memory T cells in the allogeneic TCR-dependent alloreactivity assay were overcome by IL-2.

Following CD28 co-stimulation, activation marker expression decreased slightly after treatment with sirolimus alone (naïve, 340.3%; memory, 245.6%), increased on both T-cell populations after IL-2 supplementation (naïve, 474.3%; memory, 792%), and was highest after treatment with IL-2 alone (naïve, 777%; memory, 1,477%) (Figure 1B).

Antigen-independent stimulation via the TCR using anti-CD3/CD28 beads was further assessed in order to determine if the positive effect of sirolimus depends not only on IL-2, but also on TCR-activation (Figure 1C). Sirolimus treatment did not influence the overall activation marker expression on naïve T cells. On memory T cells, the addition of IL-2 resulted in slight upregulation (408.5%), which was further increased in the presence of sirolimus (419.3%). Thus, in the absence of TCR signaling (Figure 1B), sirolimus had a negative effect on naïve and memory T cells which was compensated by IL-2 supplementation, but was still lower than with IL-2 alone. Overall, the positive effect of sirolimus plus IL-2 was highest on memory T cells. These results indicate that memory T cells are more susceptible to the immunostimulatory effect of sirolimus, and that this effect strongly depends on activation of the TCR via either allogeneic target cells (Figure 1A) or anti-CD3/CD28 (Figure 1C) as well as on the presence of IL-2.

### Sirolimus Suppresses CMV-Specific T-Cell Expansion in a Dose-Dependent Manner

**Figure 2:**
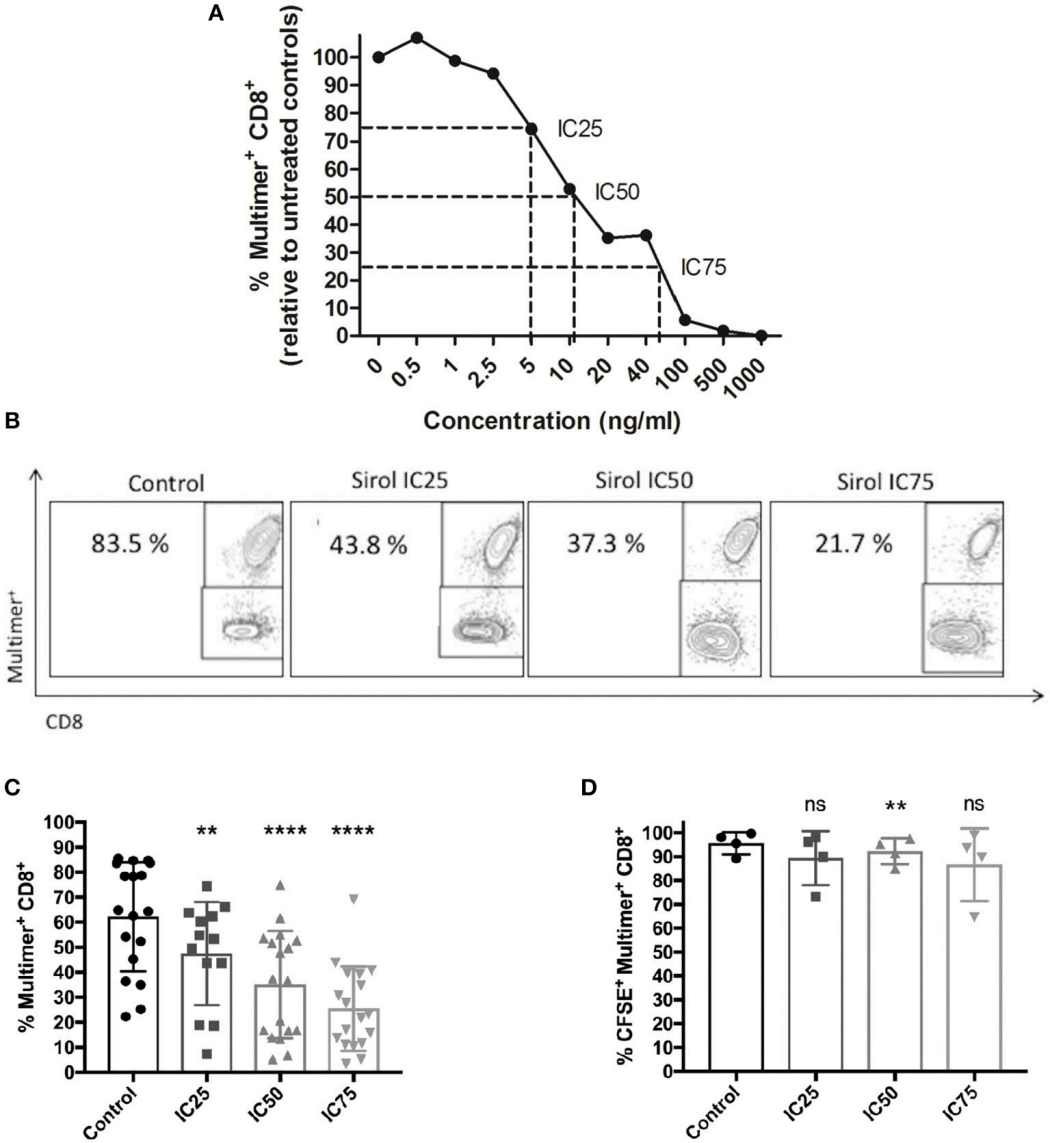
Dose-dependent suppression of CMV-specific T-cell expansion by sirolimus. **(A)** Normalized percentages (%) of CMV-specific multimer^+^CD8^+^ T cells (*n* ≥ 3) on day 7, as measured by multicolor flow cytometry. The indicated sirolimus (Sirol) concentrations (0.5–1,000 ng/ml) were added on day 0, and cells were stimulated with A02pp65_p_-loaded aAPCs in the presence of IL-2 (50 U/ml). Cultures without sirolimus served as controls. Optimal concentrations of sirolimus were defined by inhibition of CMV-specific CD8^+^ T cell expansion by 25% (IC25; 5 ng/ml), 50% (IC50; 10 ng/ml), and 75% (IC75; 40 ng/ml). **(B)** Representative dot-plot showing the percentages of expanded CMV-specific CD8^+^ T cells treated with sirolimus (from one donor). **(C)** Percentages of expanded multimer^+^ CMV-specific CD8^+^ T cells treated with sirolimus (*n* = 18). **(D)** Percentages of proliferated CFSE^+^multimer^+^ CMV-specific CD8^+^ T cells. Data are shown as means plus minus (±) standard deviation (SD). The two-paired Student's *t*-test was used to test for statistically significant differences [**p < 0.01, ****p < 0.0001, non-significant (ns)].

To investigate the effects of sirolimus on CMV-specific CD8^+^ T cells, the optimal concentration range of sirolimus for *in vitro* experiments was determined, which was set to be 0.5–1,000 ng/ml (Figure 2). As expected, sirolimus had a dose-dependently negative effect on the expansion of CMV-specific T cells. Normalized values for the generation of A02pp65_p_-specific (multimer^+^) CD8^+^ T cells (Figure 2A), 5, 10, and 40 ng/ml were determined to be the respective inhibitory concentration (IC) for 25, 50, and 75% inhibition of generation of CMV-specific multimer^+^CD8^+^ T cells relative to numbers in untreated controls (100%).

Figure 2B shows one representative result. Overall and relative to untreated controls (mean of 62.2% multimer^+^CD8^+^ T cells), 47.5, 35.1, and 25.5% multimer^+^CD8^+^ T cells were generated at IC25, IC50, and IC75 (Figure 2C). IC50 (10 ng/ml) was preferably used in subsequent experiments as it reflects the therapeutic concentration (13, 35); moreover, the number of CMV-specific T cells were generated at IC50 to perform T-cell functional assays.

To investigate whether the observed suppression of T-cell expansion was due to decreased proliferation and/or increased cell death, cell proliferation was monitored by CFSE dilution on day 7 (Figure 2D, Figure S1A), trypan blue exclusion (Figure S1B), and 7AAD staining (Figure S1C). Only a slight effect of sirolimus on the proliferation capacity of CMV-specific T cells was observed (mean IC25: 89.4%, mean IC50: 92.3%, and mean IC75: 86.6% vs. mean control value of 95.6%, Figure 2D). As expected, treatment significantly inhibited the proliferation (total number) of CD8^+^ T cells in a dose-dependent manner (Figures S1A,B), without increasing cell death (Figure S1C). Thus, sirolimus inhibits the generation frequency of CMV specific T-cells without influencing their proliferation capacity.

### Sirolimus Has No Effect on Effector Memory Phenotype but Upregulates Activation Marker Expression on CMV-Specific T Cells

**Figure 3:**
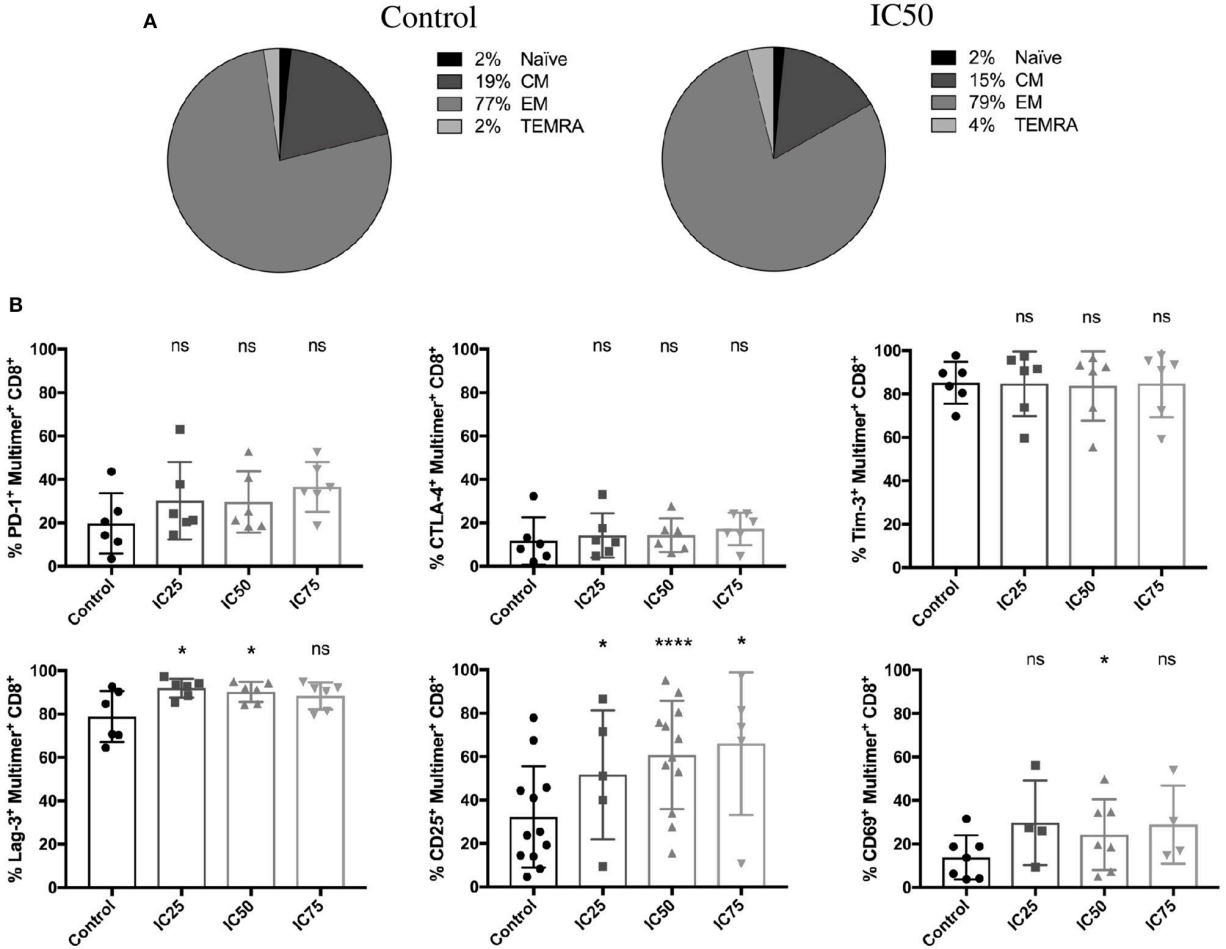
Stable effector memory phenotype and increased expression of activation markers on CMV-specific T cells after sirolimus treatment. **(A)** CMV-specific multimer^+^ T-cell phenotyping was performed using CD62L and CD46RA surface markers on day 7 after A02pp65_p_-loaded aAPC stimulation without (control) or with IC50 sirolimus treatment (IC50) in the presence of IL-2. CM = central memory T cells; EM = effector memory T cells; TEMRA = effector memory T cells expressing CD45RA determined as percentages (%) (*n* = 4). **(B)** Percentages of expression of PD-1, CTLA-4, Tim-3, Lag-3, CD25, and CD69 on CMV-specific T cells (*n* ≥ 4). Data are shown as means ± SD. The two-paired Student's *t*-test was used to test for statistically significant differences [*p < 0.05, ****p < 0.0001, non-significant (ns)].

The phenotype of CMV-specific CD8^+^ T cells generated in response to sirolimus treatment was determined based on CD45RA and CD62L expression before and after 7 days of treatment. As expected, CMV-specific (Figure 3A), and total CD8^+^ T cells (Figure S2A) were mainly effector memory (EM) T cells (CD45RA^−^CD62L^−^, multimer^+^: 79% and total CD8^+^: 64%, respectively), and their frequencies were only slightly higher than those in untreated controls (multimer^+^: 77%, total CD8^+^: 68%).

Upregulation of classical activation (CD25, CD69) and exhaustion marker (PD-1, Lag-3) expression was markedly increased after sirolimus treatment in CMV-specific T cells (Figure 3B) compared to total CD8^+^ T cells (Figure S2B). These results were not unexpected since the transient expression of exhaustion markers is also used to describe T-cell activation (37, 38). Similar to the lack of effect on the expression of CTLA-4 and Tim-3 on multimer^+^ T cells (Figure 3B), treatment had no significant effect on the overall expression of activation and exhaustion markers on total CD8^+^ T cells (Figure S2B). Interestingly, Tim-3 and Lag-3 expression on CD8^+^ T cells was significantly downregulated in response to sirolimus treatment. Taken together, these data suggest that CMV-specific T cells expand on aAPCs in the presence of IL-2 and sirolimus exhibit an effector memory-like phenotype characterized by strong expression of activation markers.

These results are in line with those shown in Figure 1 and highlight the strong immunostimulatory effects of sirolimus on memory T cells in the presence of IL-2 and TCR signaling.

### CMV-Specific T Cells Show Potent Increase in Functionality and Target Cell Recognition After Sirolimus Treatment

**Figure 4:**
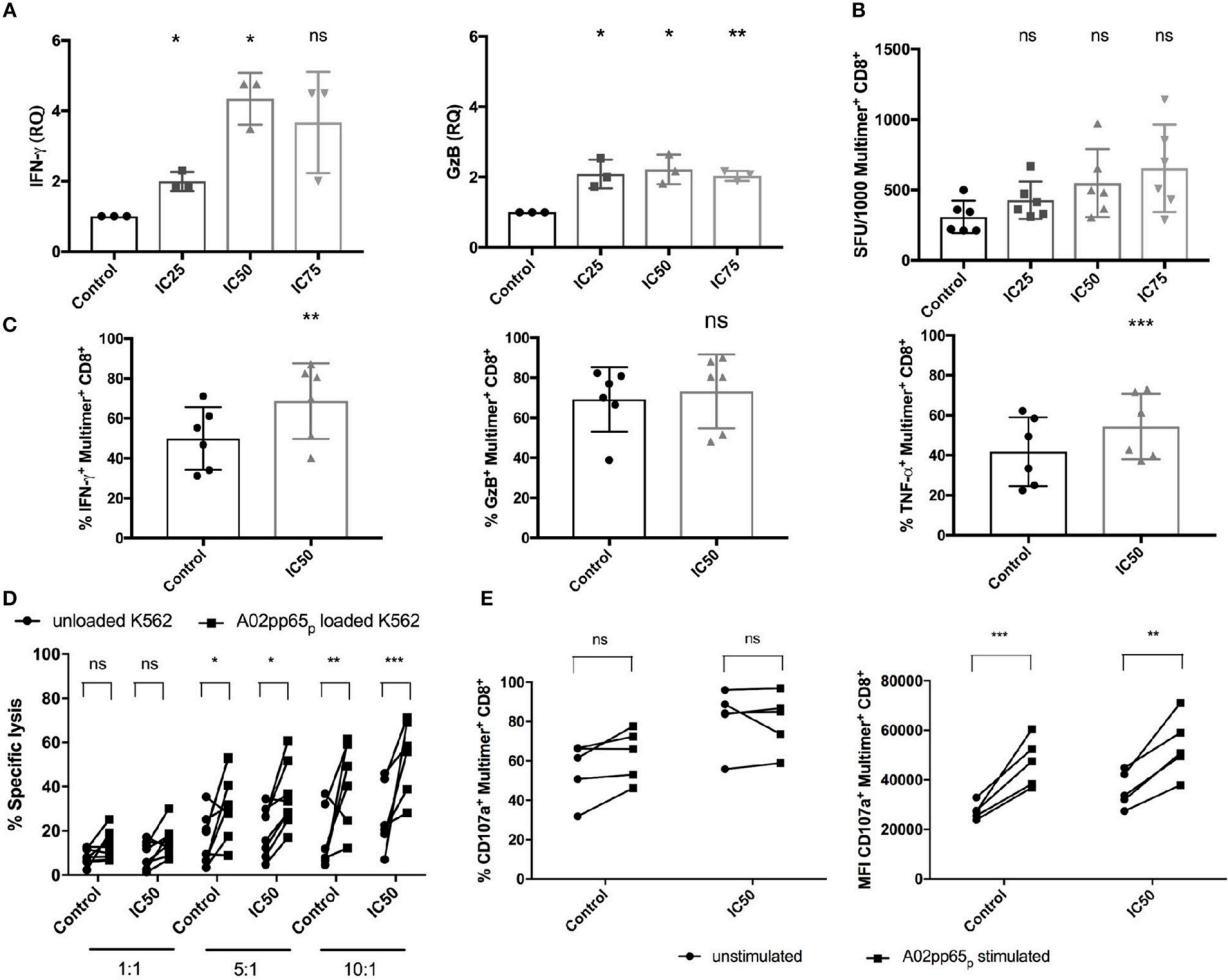
Improved functionality of CMV-specific T cells after sirolimus treatment. **(A)** Relative quantities (RQ) of IFN-γ and granzyme B (GzB) secretion were assessed using RT-qPCR following overnight A02pp65_p_ re-stimulation on aAPC stimulated and sirolimus treated and untreated CD8^+^ T cells. **(B)** IFN-γ expression levels determined by IFN-γ ELISpot assay and expressed as the number of spot-forming units (SFU) per 1,000 CMV-specific multimer^+^CD8^+^ T cells. **(C)** Percentages (%) of intracellular IFN-γ, GzB, and TNF-α on CMV-specific multimer^+^CD8^+^ T cells, as determined by intracellular staining using multicolor flow cytometry. **(D)** Target-cell recognition assay was performed on day 7. Total CD8^+^ T cells were co-cultured for 5 h with A02pp65_p_-loaded and CFSE-labeled A*02-transduced K562 target cells (squares) at effector to target ratios of 1:1 (*n* = 8), 5:1 (*n* = 8), and 10:1 (*n* = 6). Unloaded K562 cells served as controls (spheres). Percentages of dead cells were detected by 7AAD staining and multicolor flow cytometry. **(E)** Degranulation was determined as the percentage and median fluorescence intensity (MFI) of CD107a expression on CMV-specific multimer^+^CD8^+^ T cells by multicolor flow cytometry following 4 h of re-stimulation with A02pp65_p_ (*n* = 5). Values are displayed as mean (±) SD. Statistical analysis: **(A–C)** two-paired Student's *t*-test and **(D,E)** two-way analysis of variance [*p < 0.05, **p < 0.01, ***p < 0.001, non-significant (ns)].

To evaluate whether sirolimus has an influence on antigen-specific effector function (Figure 4), mRNA expression of effector molecules such as IFN-γ and GzB was measured following overnight antigen re-stimulation by reverse-transcription quantitative PCR (RT-qPCR). Sirolimus-treated cells showed significantly higher levels of IFN-γ (relative quantity (RQ) mean IC25: 1.9, IC50: 4.3, IC75: 3.6) and GzB (RQ mean IC25: 2.1, IC50: 2.2, IC75: 2) mRNA expression than untreated controls (Figure 4A).

IFN-γ ELISpot assay and combined ICS with multimer staining were done to confirm these results and further characterize the responsiveness of CMV-specific T-cell responses (Figures 4B,C, Figures S3A,B). Analysis of the total number of SFU showed that sirolimus treatment led to a slight reduction of IFN-γ secretion compared to the controls (control: mean of 509 SFU, IC25: 411, IC50: 473, and IC75: 347; Figure S3A). However, analysis of SFU per 1,000 CMV-specific CD8^+^ T cells showed increased IFN-γ expression (control: mean of 308 SFU, IC25: 427, IC50: 548, and IC75: 654; Figure 4B). Our evaluation of the effector function of CMV-specific CD8^+^ T cells by ICS (Figure 4C, Figure S3B) showed significant increases in the frequency (Figure 4C) and in median fluorescence intensity (MFI) (Figure S3B) of IFN-γ, GzB, and TNF-α secretion in sirolimus-treated virus-specific T cells. LEGENDplex™ Human CD8/NK Panel Detection Antibodies were used for further quantification of these and other effector cytokines from cell culture supernatant (Figure S3C). Overall expression of effector cytokines (e.g., IL-2, perforin etc.) was increased in sirolimus-treated cells compared to untreated controls. These results showed clear evidence of a higher functionality of sirolimus-treated CMV-specific T cells, which was further strengthened by proof of the capacity of sirolimus-treated CD8^+^ T cells to lyse A02pp65_p_-expressing K562 target cells (Figure 4D). CD8^+^ T cells generated over 7 days with and without IC50-level sirolimus treatment were co-cultured with peptide-unloaded or -loaded target cells for 5 h at the following three different effector:target (E:T) ratios: 1:1, 5:1, and 10:1. Compared to untreated cells, the capacity of sirolimus-treated CD8^+^ T cells to recognize and lyse target cells (CFSE^+^7AAD^+^) was higher at every E:T ratio and was the highest at 10:1 (Figure 4D).

Surface expression of CD107a on CMV-specific CD8^+^ T cells upon peptide re-stimulation was also measured to further analyze cytotoxicity. Compared to controls, treated cells showed increased CD107a expression in terms of both frequency and MFI (Figure 4E), with a significant difference in MFI. Taken together, these data indicate that sirolimus improves the functional quality of CMV-specific CD8^+^ T cells (Figure 4) while suppressing the expansion of CMV-specific T cells (Figure 2).

### Sirolimus Does Not Affect the Dynamics of CDR3 Repertoires in CMV-Specific T Cells

**Figure 5:**
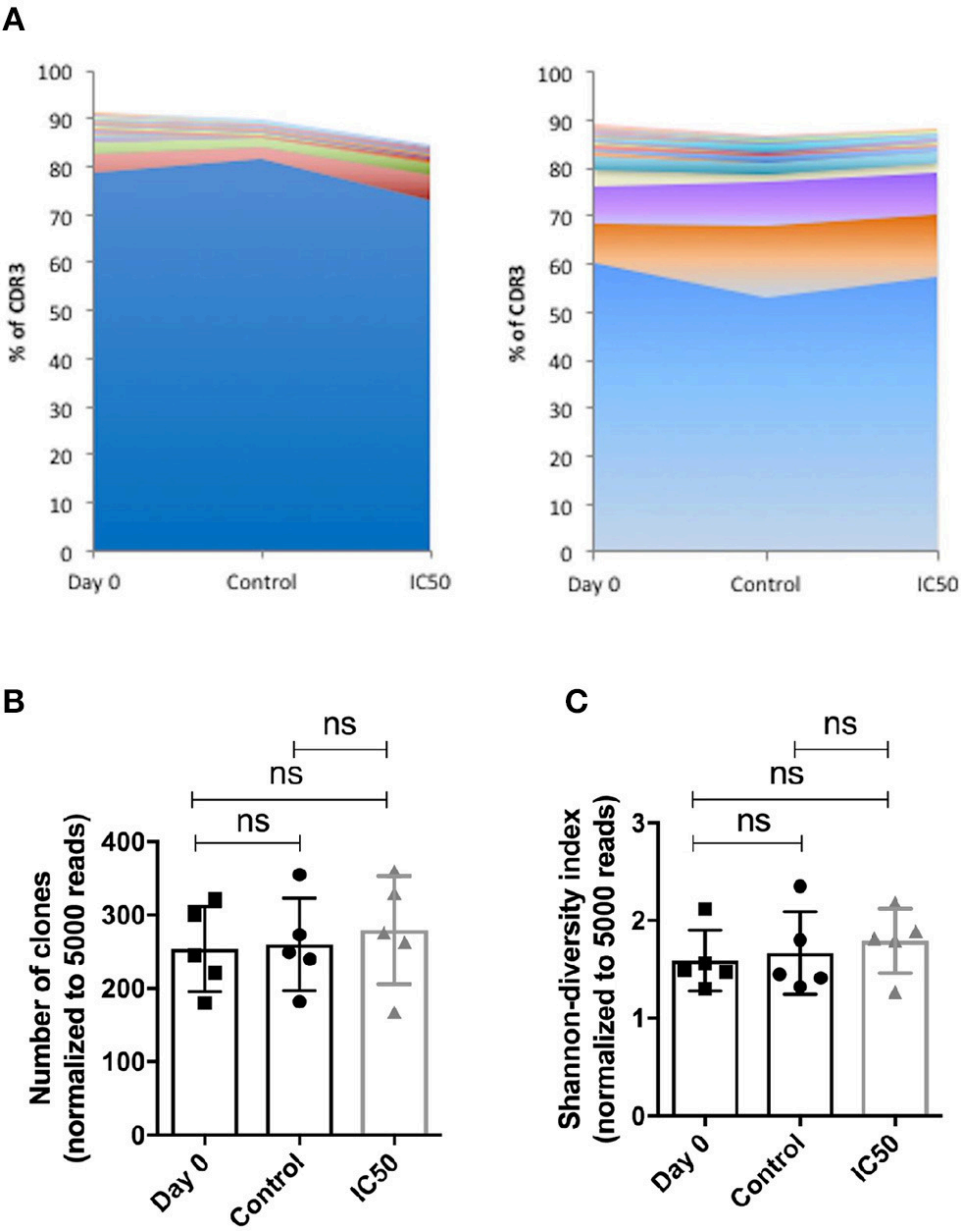
Sirolimus has no effect on the dynamics of TCR repertoires. Isolated CD8^+^ T cells were expanded for 7 days on aAPCs and treated with or without sirolimus in the presence of IL-2. On day 7, CMV-specific CD8^+^ cells were sorted based on their multimer-specificity (A02pp65_p_) by high speed flow cytometry sorters and subjected to high throughput TCR repertoire analysis. **(A)** The highest expanded CDR3 clones in two donors are shown as percentages (%) of productive reads. **(B)** Numbers of the CDR3 clones were normalized to 5,000 reads. **(C)** The Shannon index indicates the diversity of the repertoires, shown as normalized values. Values are displayed as mean (±) SD. Statistical analysis: two-way analysis of variance [non-significant (ns)].

In order to answer the question of whether sirolimus promotes the expansion of sirolimus-resistant T-cell clones, an RNA-based next-generation sequencing (NGS) approach was applied to monitor the dynamics of TCR β-chain repertoires in multimer-sorted CMV-specific CD8^+^ T cells on day 7 (Figure 5). As expected, the pre- and post-expansion TCR repertoires were highly clonal independently of treatment, and they consisted of a very small number of expanded clones as displayed in stacked area graphs in Figure 5A. Likewise the clonal sizes between repertoires were highly similar (Figure 5B). Interestingly, none of the most frequent CDR3 sequences were shared by the analyzed healthy donors (as indicated by the color code and displayed CDR3 sequences in Figure 5A). Overall, diversity was low and did not change after expansion and treatment (Figure 5C). Taken together, RNA-based NGS and CDR3 based analysis reflected no change in the T-cell repertoire post-sirolimus treatment, which significantly argue against effect of sirolimus on any T-cell clone. These results suggest that the αβ TCR repertoire reflects the immunological history of an individual rather than the selective pressure of immunosuppression on a healthy individual.

### Signaling Pathways Involved in T-Cell Activation and Function Are Differently Regulated by Moderate mTORC1 Inhibition

**Figure 6:**
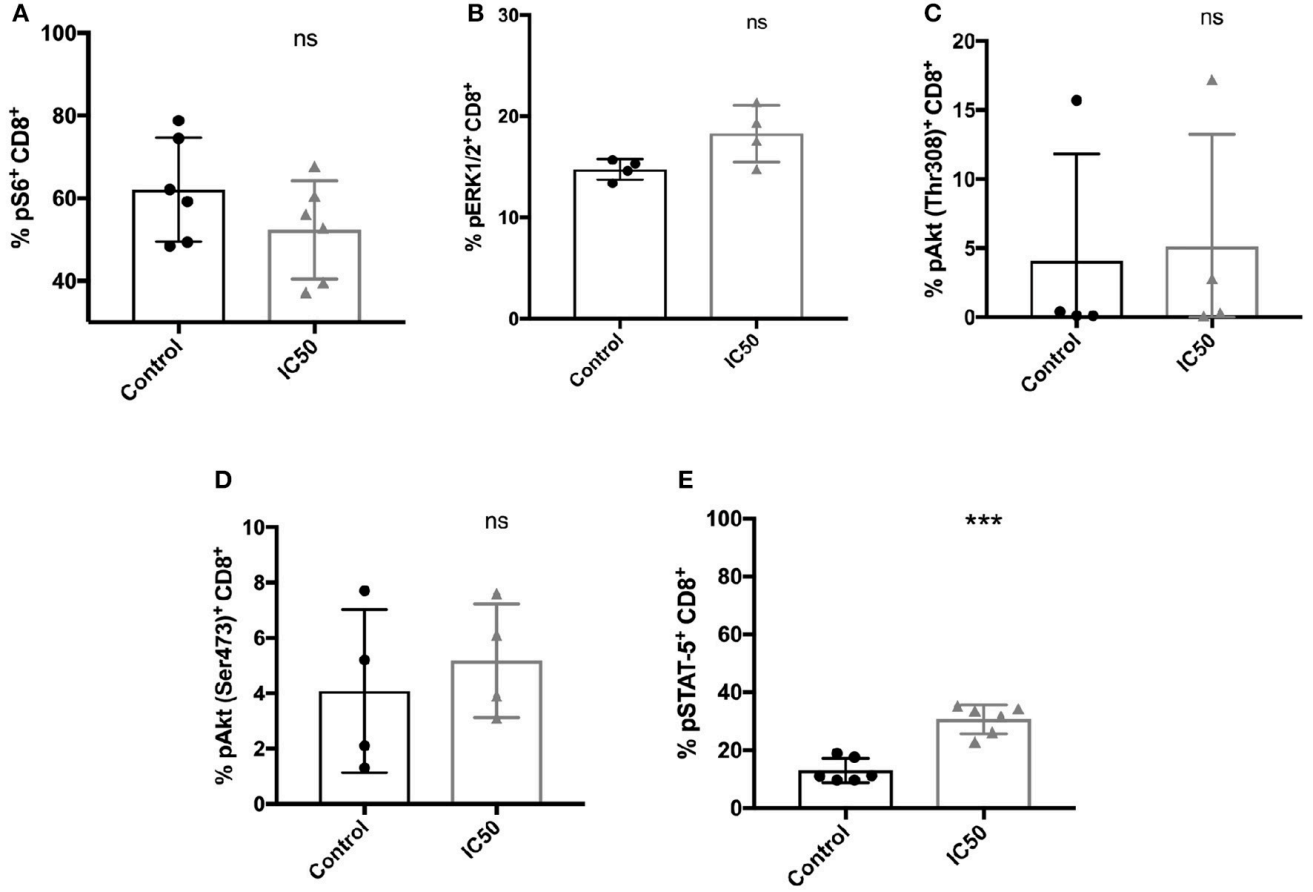
Incomplete mTORC1 inhibition has differential effects on the phosphorylation of signaling molecules. Total CD8^+^ T cells were re-stimulated with 10 μg/ml A02pp65_p_ for 1 h following 7 days of aAPC stimulation with or without sirolimus in the presence of IL-2, and the percentages (%) of phosphorylated **(A)** S6, **(B)** ERK1/2, **(C)** Akt^Thr308^, **(D)** Akt^Ser473^, and **(E)** STAT-5 were determined by phospho-flow cytometry. Values are displayed as mean (±) SD. The two-paired Student's *t*-test was used to test for statistically significant differences [***p < 0.001, non-significant (ns)].

The mechanism of sirolimus to improve the functionality of CMV-specific CD8^+^ T cells was evaluated by analyzing the phosphorylation of kinases such as Akt and ERK1/2, respectively, and of the proteins (S6 and STAT-5) which are involved in T-cell activation and signaling (Figure 6; Figure S4). Antigen-specific phosphorylation of S6, a downstream target of mTORC1 was evaluated to confirm the inhibitory effect of sirolimus on mTORC1. Since, the IC50 was used in order to generate a sufficient number of CMV-specific T cells for further functional analysis, incomplete inhibition of mTORC1 was expected. The frequency of phosphorylated S6 was reduced in sirolimus-treated cells (mean 52.3%, Figure 6A), but MFI analysis (mean 7092.5, Figure S4A) showed that phosphorylation was higher in treated cells compared to untreated controls (62.1%, 4785.5). Treated CD8^+^ T cells showed a TCR responsiveness gain compared to controls, as reflected by increased frequency and MFI values for phosphorylation of distal signaling molecules such as ERK1/2, Akt^Ser473^, and Akt^Thr308^ (Figures 6B–D, Figures S4B–D). These results are in line with the detected increase in CD25 (also known as IL-2R alpha chain) expression on sirolimus-treated cells (Figure 1, Figure 3B), and they might indicate a shift toward IL-2-dependent regulation of CMV-specific CD8^+^ T-cell development. Indeed, sirolimus treatment resulted in significant increases in STAT-5 phosphorylation in terms of frequency (Figure 6E, 30.6 vs. 12.9%) and MFI values (Figure S4E, 2098.3 vs. 1,424), and functional improvement of CMV-specific T-cells.

### Sirolimus-Induced Functional Improvement Correlates With IL-2R Activation on CMV-Specific T Cells

**Figure 7:**
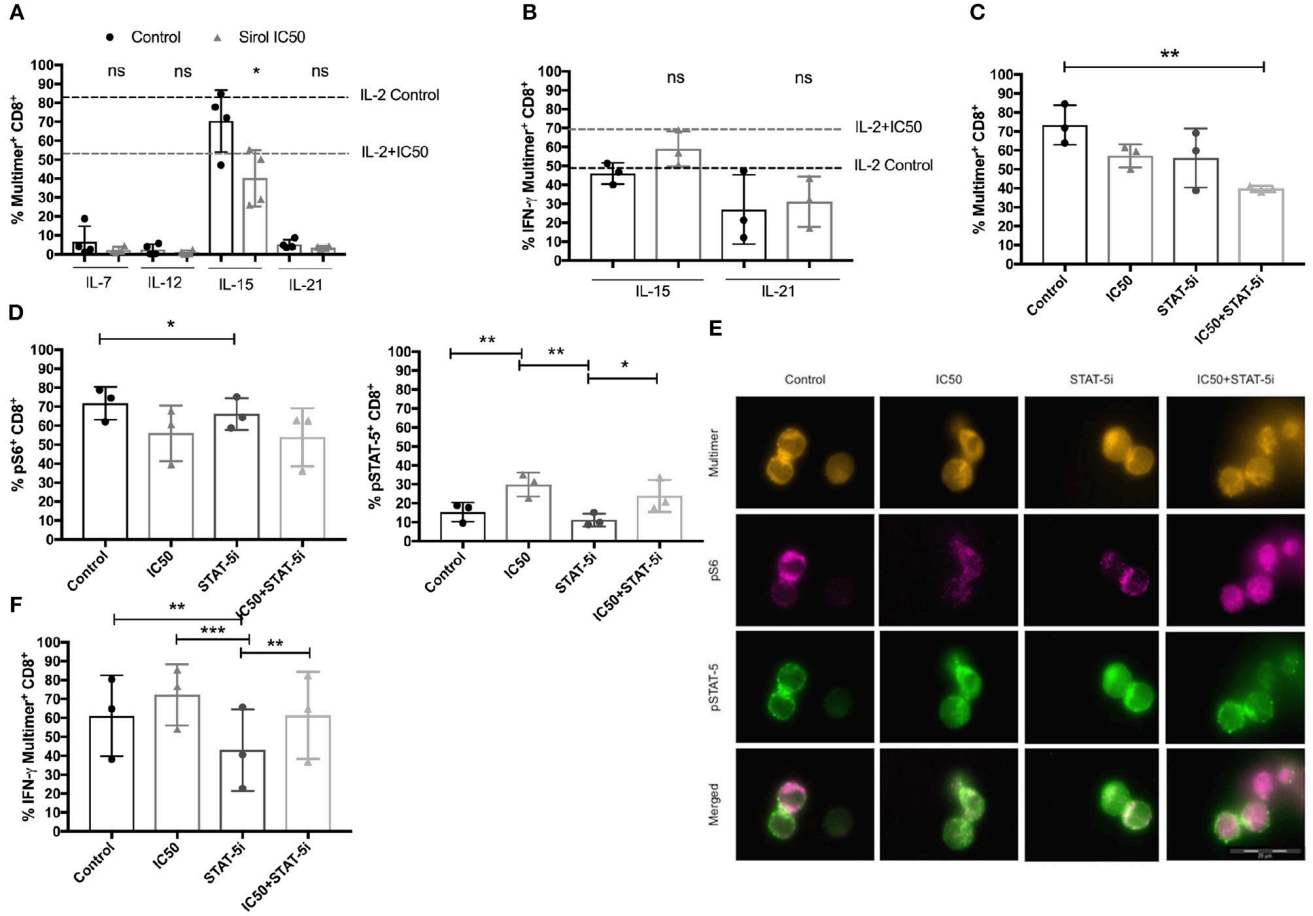
Functional improvement of CMV-specific T cells treated with sirolimus strongly depends on IL-2R-driven STAT-5 signaling. **(A)** Percentages (%) of CMV-specific multimer^+^CD8^+^ T cells after 7 days of aAPC stimulation with or without sirolimus in the presence of the following cytokines: IL-7, IL-12, IL-15, IL-21 (each 10 ng/ml) which were added independently as different sets of cell cultures. Results for supplemental IL-2 (50 U/ml) without sirolimus (black dashed lines) and with sirolimus (gray dashed lines) are shown (*n* = 4). **(B)** Percentages of IFN-γ following 5 h peptide re-stimulation, as determined by intracellular cytokine staining on CMV-specific multimer^+^CD8^+^ T cells (*n* ≥ 3). **(C)** Percentage of expanded CMV-specific multimer^+^CD8^+^ T cells following overnight STAT-5 inhibition (STAT-5i) on day 7. **(D)** Percentages of pS6 and pSTAT-5, as determined by phospho-flow cytometry or **(E)** fluorescence microscopy on CMV-specific CD8^+^ T cells after 1 h of peptide re-stimulation. **(F)** Intracellular expression of IFN-γ (%) following 5 h of peptide re-stimulation, as measured by multicolor flow cytometry. Values are displayed as mean (±) SD. Statistical analysis: **(A,B)** Student's *t*-test and **(C,D,F)** two-way analysis of variance (*p < 0.05, **p < 0.01, ***p < 0.001).

Next, to determine if IL-2, IL-7, IL-12, IL-15, and IL-21 can differentially affect the sirolimus-related expansion and functionality of CMV-specific T cells, these immune regulatory cytokines were added to the culture media independently as a different sets of cell cultures (Figures 7A,B, Figure S5A). The results showed that IL-7, IL-12, and IL-21 are not as essential as IL-2 and IL-15 for CMV-specific T-cell expansion (Figure 7A). In particular, the expansion of multimer^+^CD8^+^ T cells was barely affected by these cytokines (frequencies < 10%). Only the addition of IL-2 and IL-15 resulted in T-cell expansion, which was further impaired by sirolimus treatment (IL-2 control: 82.5% and IC50: 54.8%, IL-15 control: 70.4%, and IC50: 40.2%). Upon evaluating the effector function of CMV-specific CD8^+^ T cells by ICS, we observed an overall increase in IFN-γ expression (Figure 7B, Figure S5A) after sirolimus treatment in the presence of IL-15. The tendency observed with IL-2 and IL-21 was comparable to that in untreated controls. However, the responses observed with IL-2 or IL-15 were more robust in terms of the total number of cells (data not shown) and CMV-specific CD8^+^ T-cell responses determined by multimer and intracellular staining (Figures 7A,B, Figure S5A). Increased expression of IFN-γ corresponded to an overall increase in capacity of sirolimus-treated CMV-specific CD8^+^ T cells to recognize and lyse A02pp65_p_-loaded K562 target cells and to the expression of CD107a in the presence of IL-15 (Figures S5B,C), although these responses were stronger with IL-2 and sirolimus (Figures 4D,E). These results indicate that IL-2R subunit-sharing cytokines, particularly IL-2 and IL-15, support antiviral T-cell responses during mTORC1 inhibition by sirolimus.

### Sirolimus Treatment and STAT-5 Inhibition Impair the Quality and Quantity of CMV-Specific CD8^+^ T cells

Sirolimus-treated CMV-specific CD8^+^ T cells were further tested for differences in functionality and S6 and STAT-5 phosphorylation after overnight STAT-5 inhibition to prove the beneficial effect of STAT-5 on functionality (Figures 7C–F). Following short STAT-5 inhibition, CD8^+^ T cells displayed a decreased frequency of expanded CMV-specific multimer^+^ T cells in comparison with cells treated with and without sirolimus alone or STAT-5 inhibitor alone in the presence of IL-2 (Figure 7C) or IL-15 (Figure S5D). The frequency of pS6 (Figure 7D) on CD8^+^ T cells and its localization on multimer^+^CD8^+^ T cells was lower after combined treatment as compared to other conditions (Figure 7E). No differences could be seen in phosphorylation of S6 in cells treated with IL-15 (Figure S5E).

Reduced antigen-specific expression of IFN-γ was observed following inhibition of STAT-5 alone or with sirolimus in combination with either IL-2 (Figure 7F) or IL-15 (Figure S5F). This indicated a decrease in STAT-5 function, which was further confirmed by decreased phosphorylation of STAT-5 in cells treated with either IL-2 (Figures 7D,E) or IL-15 (Figure S5E). However, IFN-γ and pSTAT-5 expression remained higher in cells treated with the combination of sirolimus and STAT-5 inhibitor than in untreated controls; expression levels of both were moderately inhibited and showed the same tendency as in cells treated with sirolimus alone. Collectively, these data suggest that IL-2R-driven STAT-5 signaling plays a major role in the improvement of antiviral T-cell responses during mTORC1 inhibition.

### Sirolimus Differently Affects Expression of miRNAs and Effector/Target Genes

**Figure 8:**
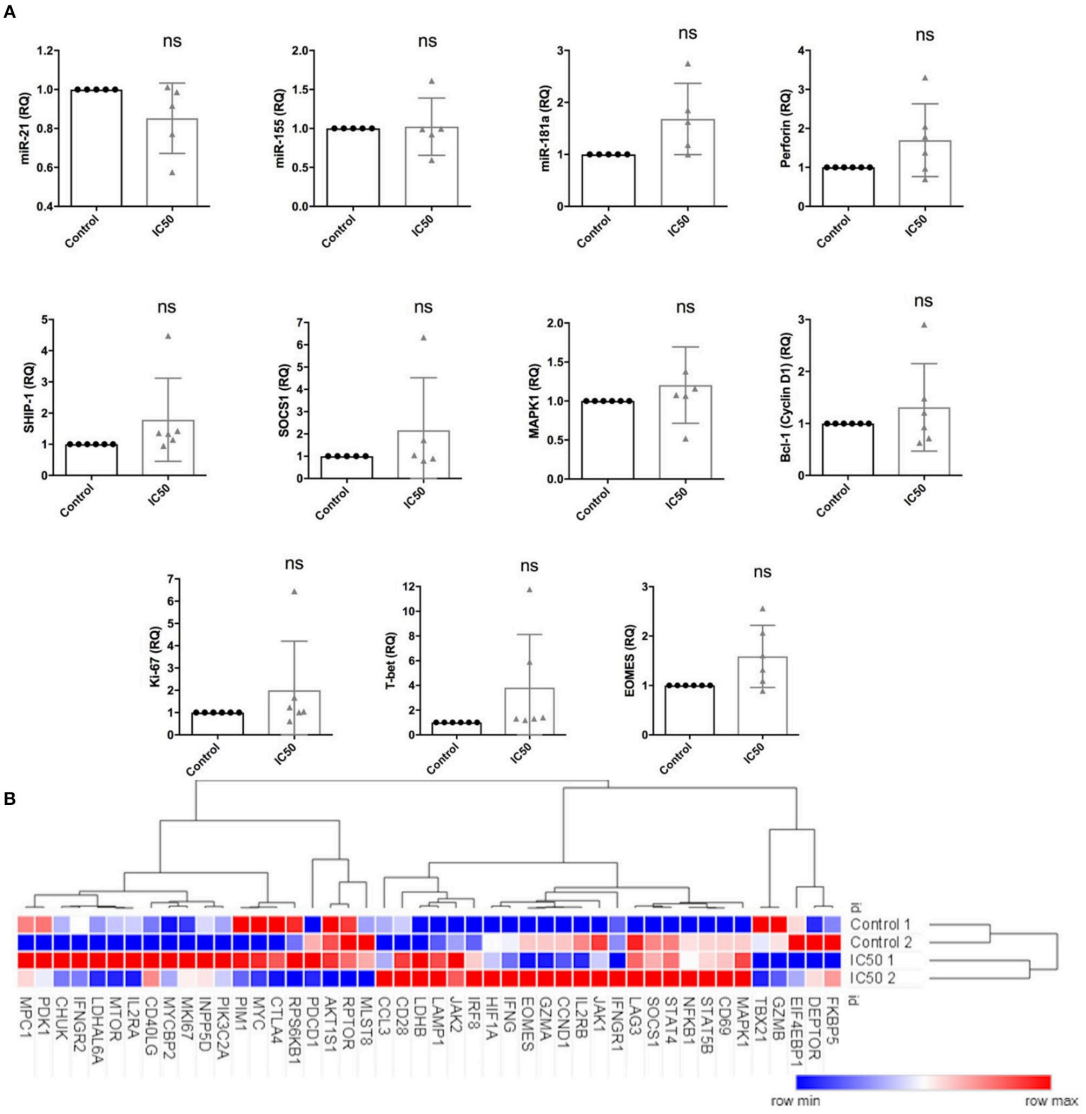
Sirolimus differentially affects the expression of target and effector genes and miRNAs. Expression levels of **(A)** miR-21, miR-155, miR-181a, perforin, SHIP-1, SOCS1, MAPK1, Bcl-1, Ki-67, T-bet and EOMES, as determined by RT-qPCR following overnight A02pp65_p_ re-stimulation on 7 day aAPC stimulated CD8+ T cells treated with or without sirolimus in the presence of IL-2. **(B)** Selected results from global gene expression analysis in multimer-sorted CMV-specific CD8^+^ T cells on day 7 obtained from untreated (Control 1 and 2) and sirolimus-treated cells (IC50 1 and 2) of two donors (*n* = 2). CD8^+^ T cells were sorted based on their multimer specificity using high-speed flow cytometry sorters. Following overnight A02pp65_p_ re-stimulation, total RNA was isolated and investigated by microarray analysis. Clustering and heat map analyses were performed using the Morpheus web-based tool. The data are means ± SD. The two-paired Student's *t*-test was used to test for statistically significant differences [non-significant (ns)].

Expression of miR-21, miR-155, and miR-181a was studied since these miRNAs are known to be involved in the regulation of T-cell activation and function as well as in the expression of several target and effector molecules, such as perforin, Bcl-1, SOCS1, SHIP-1, T-bet, MAPK1/ERK, EOMES, and Ki-67. Expression levels were analyzed by RT-qPCR following overnight antigen re-stimulation on sirolimus treated or untreated CD8^+^ T cells (Figure 8A).

Sirolimus reduced miR-21 expression but did not affect miR-155 (Figure 8A). Interestingly, while both miR-155 and miR-181a target SHIP-1 and SOCS1, miR-155 was not affected. Sirolimus treatment resulted in increased expression of miR-181a, perforin, MAPK1, Bcl-1 (one of the main targets of mTORC1 and STAT-5 signaling), Ki-67, T-bet and EOMES on CD8^+^ T cells (Figure 8A). RNA-based microarray analysis was subsequently performed to confirm the RT-qPCR results and to investigate how these and other key genes are regulated in multimer-sorted CMV-specific T cells. Similar gene expression profiles were observed on treated and untreated multimer-sorted antigen-specific CD8^+^ T cells following antigen re-stimulation (Figure 8B). Genes coding proteins in mTORC1 were clearly downregulated by sirolimus (FKBP5, DEPTOR), whereas those involved in IL-2R pathways (IL2R, JAK1, STAT5B, IFNG) were upregulated. Although these results support our hypothesis that the improved functionality of CMV-specific T cells strongly depends on the presence of TCR activation, co-stimulation and IL-2, further investigation is necessary to better understand and obtain more insight into this highly complex network regulating CD8^+^ T-cell biology.

### Improvement of Functional Responses of CD8^+^ T-Cells Treated With Sirolimus *in vivo* Strongly Depends on the Presence of TCR Activation, Co-stimulation and IL-2

**Figure 9:**
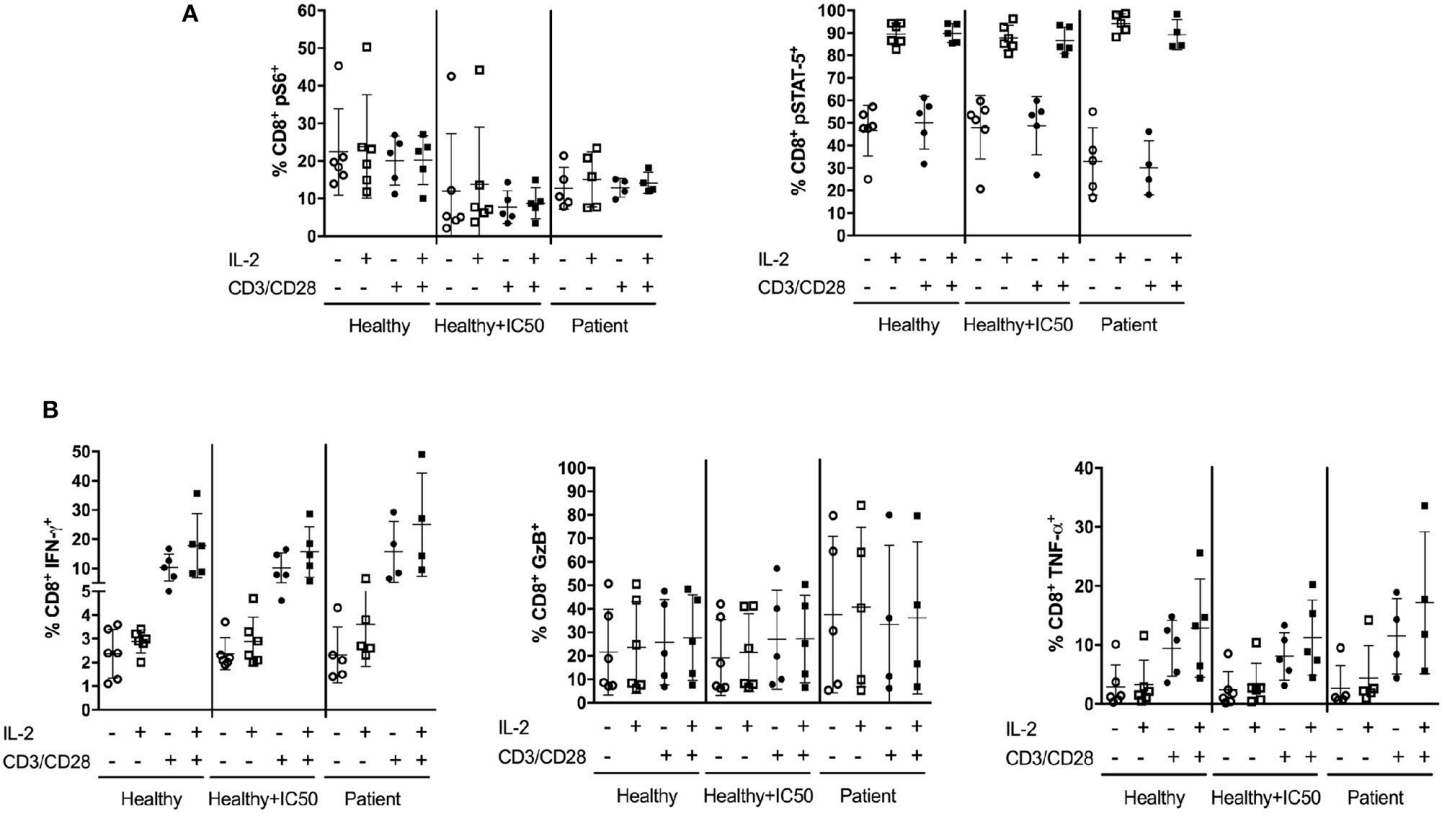
*In vitro* IL-2 treatment with TCR activation increases CD8^+^ T-cell responses after *in vivo* sirolimus treatment. PBMCs from patients (*n* ≥ 4) treated with sirolimus *in vivo*, PBMCs from untreated healthy donors (*n* ≥ 5) treated overnight with or without sirolimus (IC50) *in vitro* were stimulated with (+) or without (–) IL-2 (100U/ml) in the presence (+) or absence (–) of CD3/CD28 beads. **(A)** Following 15 min of stimulation, the percentages (%) of pS6 and pSTAT-5 were determined by phospho-flow cytometry. **(B)** Percentages of intracellular expression of IFN-γ, GzB and TNF-α in CD8^+^ T cells were measured by multicolor flow cytometry after 5 h of stimulation. The data are shown as mean ± SD. Statistical analysis was done by two-way analysis of variance. No significant differences were determined.

To further investigate the impact of sirolimus and IL-2, we analyzed peripheral blood mononuclear cells (PBMCs) from sirolimus-treated patients for the phosphorylation of S6 and STAT-5 and for the expression of effector cytokines such as IFN-γ, GzB, and TNF-α following short-term polyclonal CD3/CD28 stimulation in the presence or absence of IL-2 and compared to controls (PBMCs from healthy donors). As expected, sirolimus treatment downregulated S6 phosphorylation in patients and healthy controls compared to levels in untreated healthy controls (Figure 9A). Although, stimulation with IL-2 alone led to an only slight increase in pS6 frequencies, the same tendency was observed in all groups. In contrast, stimulation with CD3/CD28 had no effect. Overall, pS6 was lower in patients and only slightly impaired in healthy donors treated with sirolimus than in untreated controls. Phosphorylated STAT-5 (Figure 9A) was slightly higher in healthy controls with or without sirolimus than in patients treated with sirolimus. IL-2 alone or in combination with CD3/CD28 cross-linking always resulted in the upregulation of pSTAT-5 frequencies; the highest in *in vivo* treated sirolimus patients with IL-2 alone was observed. Although no differences in IFN-γ, GzB, or TNF-α expression were measured between the *in vitro* treated and untreated groups, IL-2-specific production of these cytokines was slightly higher (Figure 9B), and was increased further by CD3/CD28 stimulation compared to unstimulated controls and to *in vivo* sirolimus treated patients. Taken together, these results indicate that IL-2 increases the functionality of CD8^+^ cells treated with sirolimus *in vivo*, and that this effect can be further enhanced via TCR activation.

## Discussion

The protein kinase mTOR acts as a multichannel processor in a cellular-nutrient-sensing network and plays a major role in integrating diverse environmental signals that affect immune cell growth, proliferation and function. The inhibition of mTOR signaling by the immunosuppressive drug sirolimus is an established therapeutic strategy in transplantation medicine (1, 39). On the one hand, only moderate effects of sirolimus monotherapy in preventing graft rejection and GvHD have been observed in the transplant setting. On the other hand, various investigators have shown that transplant patients treated with this mTOR inhibitor or got everolimus-based immunosuppressive regimen have better control of pathogen infections or reactivations and, therefore, have better clinical outcomes (1, 12, 18–25, 40).

This comprehensive study provides insight into the paradoxical effect of sirolimus on naïve and CMVpp65-specific CD8^+^ memory T cells generated by a unique aAPC-based assay. We investigated the effects of TCR signaling and co-stimulatory signals and the role of mTORC1 signaling on memory T cells, focusing on fundamental elements of T-cell function and on the diversity of TCR repertoire, activation of signaling pathways, and expression of target and effector molecules. Surprisingly, we found that sirolimus–treated cells are not only functional but also have significantly better function than untreated controls. The paradoxical effect of sirolimus strongly depends on the presence of TCR activation with co-stimulation and IL-2.

As expected and in contrast to memory T-cell activation, the activation of naïve T cells was lower in response to allogeneic TCR-dependent stimulation, antigen-independent TCR stimulation via CD3/CD28 crosslinking, and co-stimulatory signals alone and in the presence of IL-2. These results underline the rationale behind using immunosuppressive agents such as sirolimus to prevent graft rejection and GvHD, as these transplant complications are caused by alloreactive cells residing within the naive CD45RA^+^ T-cell compartment (41, 42).

According to recent findings, the immunostimulatory effect of sirolimus on memory antigen-experienced T cells is strongly dependent on the presence of TCR and co-stimulatory signals and can be enhanced by IL-2. To further investigate the mode of action of sirolimus on memory T cells, a CMV-specific T-cell generation model was chosen that allowed to investigate direct effects on those cells. Bead-based aAPCs allowed for very robust expansion of antigen-specific memory T cells by providing signals for TCR recognition via HLA-A^*^02:01-CMVpp65 peptide complexes and co-stimulation through anti-CD28 monoclonal antibodies (mAbs) (33). Despite a decrease in the overall T-cell proliferation rate, we observed a strong increase in the frequency of CMV-specific multimer^+^ T cells, which was of course lower in the sirolimus-treated cells than in the untreated controls. This finding highlights the selective direct positive effect of sirolimus on antigen-specific T cells and the negative effects on those cells which were not specifically activated. The dose-dependent negative impact of sirolimus on CMV-specific T-cell expansion was counterbalanced by significant improvement in effector cell functionality, as determined by antigen-specific cytokine release (IFN-γ, GzB, and TNF-α) and cytotoxicity assays. Increased expression levels of classical activation markers such as CD25 and CD69 and transient expression of the classical exhaustion markers PD-1 and Lag-3 on CMV-specific T cells further strengthen the hypothesis that sirolimus has a positive immunostimulatory effect on antiviral T-cell functionality (37, 38). As expected, mTORC1 inhibition had no effect on the effector memory phenotype (CD45RA^−^CD62L^−^).

Although our results strongly underline earlier *in vitro* and *in vivo* observations (12, 18, 19, 23), this is the first work that shows that sirolimus has a direct immunostimulatory effect on the functionality of human CMV-specific CD8^+^ T cells. Surprisingly, we found that sirolimus not only left the treated cells functional, but also significantly improved their functionality compared to that of untreated controls. Activation that allows T cells to proliferate and develop into effective antiviral CTLs relies on four essential signals from the following sources: (1) TCR stimulation, (2) co-stimulation, (3) cytokines, and (4) chemokines (1, 43). Previous studies have shown that stimulation of TCR and CD28 in resting T cells results in IL-2-driven proliferation through the activation of phosphatidylinositide 3-kinase (PI3K) and mTOR (14). The mTORC1 inhibition with sirolimus blocks not only signals 1 and 2 but also signal 3 by attenuating IL-2R signaling, thereby preventing the full activation of T cells and the optimal expression of cyclins. Conversely, recent studies revealed that the immunosuppressive effect of sirolimus on T-cell proliferation can be diminished if those signals occur (25, 26, 29). A study of sirolimus-treated T cells by Colombetti et al. nicely showed that CD3/CD28 and IL-2/IL-2R pathways for antigen-independent TCR recognition independently regulate T-cell proliferation in sirolimus-treated T cells, that both pathways are controlled to a different extent by PI3K and mTOR, and that the CD3/CD28-driven activation of T cells is abolished in the absence of IL-2. In contrast, IL-2 induced T-cell proliferation was independently regulated by these signaling molecules (44). The present study showed that T-cell activation is influenced in the same way because the immunosuppressive effect of sirolimus can only be overcome by antigen-independent TCR stimulation via CD3/CD28 co-crosslinking and IL-2.

In 2004, Slavik et al. postulated that the functional outcome of antigen-specific memory T cells is associated with strong signals due to high affinity TCR and CD28 (26, 29). Their findings are substantiated by our results suggesting the antigenic stimulation of high-affinity HLA-A^*^02:01-restricted CMV-specific TCR and co-stimulation signals from aAPCs. In addition, IL-2 converted the sirolimus-resistant functionality of T cells in our tests using an antigen-specific and CD3/CD28 crosslinking approach. To our knowledge, this study is the first showing that sirolimus has a direct selective immunosuppressive effect on naïve and immunostimulatory effect on antigen-specific T cells.

In addition, our results showed that mTORC1 inhibition with sirolimus during aAPC stimulation results in unexpectedly higher levels of CD25 (IL-2R) expression. Therefore, it is likely that the antigen-specific T cells became more susceptible to IL-2 or IL-2R subunit-sharing cytokines (e.g., IL-15), which are known to be fundamental in the maintenance and differentiation of effector T cells (45, 46). Moreover, the addition of IL-15 resulted in the same positive effect on T-cell functionality in the presence of sirolimus, whereas supplementation with IL-7, IL-12, or IL-21—cytokines known to improve T-cell function—did not overcome the immunosuppressive effect of sirolimus in our study. Antigen-independent analysis of the T-cell functionality of samples from patients treated with sirolimus and healthy control confirmed this positive effect of IL-2.

In order to generate sufficient numbers of CMV-specific T cells to perform functional assays, we opted for incomplete inhibition of T-cell function at the IC50 level (10 ng/ml), which is approximately equivalent to the typical circulating level of the drug in immunosuppressed patients. Therefore, we expected the phosphorylation of S6, a downstream target of sirolimus-sensitive mTORC1, to occur following stimulation. Given that the drug induced moderate phosphorylation of pS6 in addition to significantly higher phosphorylation of STAT-5, a main downstream target of IL-2, it is tempting to speculate that sirolimus activates at least one more downstream pathway that enables T cells to escape cell cycle arrest and thus maintain their effector and cytotoxic functions. This hypothesis was supported by findings of increased expression of Bcl-1 and higher phosphorylation of ERK1/2 and Akt, and was further strengthened by the findings observed after inhibition of STAT-5. In conclusion, we found that the interactions of mTORC1 and IL-2R-driven STAT-5 signaling influenced the immune balance by modulating the expansion and functionality of CMV-specific T cells. To our knowledge, this is the first study investigating the interplay between mTORC1 and IL-2R-driven STAT-5 signaling in antiviral human CMVpp65_p_-specific CD8^+^ T cells.

To gain deeper insight into the mechanism of action of this mTOR inihibitor, we further investigated the mTOR inhibitor on miRNA-mediated regulation of CMV-specific T-cell function in cells treated with sirolimus. Stimulation via TCR and nuclear factor kappa-light-chain-enhancer of activated B cells (NF-κB) signaling normally induces the expression of miR-21, miR-155, and miR-181, all of which are known to promote T-cell proliferation, survival and effector function (47). As sirolimus did not affect miR-155 expression in this study, the increase in the expression of the target genes SOCS1 and SHIP1 might be related to the slight downregulation of miRNA-21. Considering these results, we would expect sirolimus-treated cells to show less functionality. On the other hand, increased miR-181a expression might be responsible for the observed increase in T-cell functionality. The expression of markers such as MAPK1, CD28, and CD40LG as determined by microarray analysis and the increased secretion of effector molecules such as IFN-γ and GzB support this idea. Further studies using anti-miRNA oligonucleotides to neutralize miRNA function will help to gain more insight into the highly complex network regulating CD8^+^ T-cell biology via miRNAs and their counterparts (48).

Recent findings demonstrated the ability of mTOR to interpret signals in the immune microenvironment and to program the generation of effector vs. memory CD8^+^ T cells via the direct line between metabolism and function (14). Our microarray analysis of multimer-sorted CMV-specific CD8^+^ T cells confirmed this, with results showing that sirolimus induces the upregulation of genes involved in glycolysis, such as CD28, AKT, and HIF1A, which promote increases in glucose uptake, PDK1, which increases the conversion of pyruvate into lactate, and upstream glycolytic enzymes such as LDH and MYC. These results are consistent with the findings of another study demonstrating that the functionality of effector CD8^+^ T cells is relies on glycolysis (49). In addition, it underlines the immunostimulatory effect of sirolimus on antiviral T-cell responses by modulating multiple environmental cues during antiviral T-cell expansion under the influence of mTORC1 inhibition.

Sequencing of the TCR β-chain repertoire of sorted CMV-specific T cells was performed to answer the question whether sirolimus–resistant clones may serve as a reservoir of shared and functional T cells and expand during immunosuppression. The ability of the adaptive immune system to respond to a wide variety of pathogens depends on the presence of a unique TCR repertoire reflecting the initial V(D)J recombination events shaped by the selection of self and foreign antigens presented by HLA molecules on APCs (50). We observed no selective effect of sirolimus on the highly unique clonal HLA-A^*^02:01-restricted CMV-specific TCR β-chain repertoires. Interestingly, no sharing of the TCRs between the analyzed healthy individuals was observed. We hypothesize that the αβ T cell repertoire reflects the individual history of immunological exposure, which is driven by the environmental milieu and not by the selective pressure of immunosuppression on a healthy individual. Further independent analysis on the TCR α-chain would be essential to complete these study findings and strengthen this hypothesis. Although we did not observe any effect of sirolimus on the TCR repertoires examined in this study, next-generation sequencing offers the possibility to identify drug-resistant TCR clones in patients who develop primary infection or reactivation during immunosuppressive treatment. Furthermore, such analyses will help to identify the dynamics of both αβ and γδ TCR repertoires in immunosuppressed recipients with viral complications treated with various immunosuppressive (51, 52).

The effect of sirolimus on human cells is even wider than previously expected. In this study, we show for the first time that sirolimus acts selectively on naive and memory T cells and directly on CMV-specific CD8^+^ T cells to promote their responses to antigens. These results clearly demonstrate the benefits of sirolimus treatment for transplant patients. On the one hand, sirolimus suppresses alloreactive naïve T cells and thereby prevents the development of GvHD. On other hand, it increases the functionality of antigen-specific T cells and might therefore promote graft-vs.-infection (GvI) and graft-vs.-tumor (GvT) responses. A better understanding of the complex effects of immunosuppressive drugs that regulate effector cell functions should provide new opportunities to further individualize immunosuppressive therapy in patients with an increased risk of viral infection and/or reactivation (53). In addition, mTOR inhibition in combination with the modulation of environmental cues using agents such as IL-2 might lead to new strategies for the treatment of infectious diseases or immunosuppressive tumors.

## Author Contributions

SB helped to design the study, performed the analyses, carried out the T-cell stimulation experiments and functional assays for healthy donors and patients, did the data generation and statistical analysis, and wrote the manuscript. ST assisted with assessing the immunofluorescence staining and microscopy, contributed helpful discussions and aided to draft the manuscript. AD helped with performing the alloreactivity and CD3/CD28 crosslinking approaches and aided to draft the manuscript. SR carried out NGS experiments, contributed helpful discussion with respect to the NGS. LP provided patient material and aided to draft the manuscript. CK helped by contributing clinical requirements and discussions and aided to draft the manuscript. MO contributed helpful and critical discussions with respect aAPC approach and helped to draft the manuscript. RB contributed helpful and critical discussions, helped to draft the manuscript, and approved the final version of the manuscript for publication. BM-K helped to design the study, provided patient material, helped by contributing critical and valuable discussions about clinical background issues and by drafting the manuscript. BE-V conceived the study, participated in its design and coordination, designed the T-cell stimulation assays, immunoassays, and data analysis procedures, and co-wrote the manuscript.

### Conflict of Interest Statement

The authors declare that the research was conducted in the absence of any commercial or financial relationships that could be construed as a potential conflict of interest.
