# Supplementary material for: Selective Effects of mTOR Inhibitor Sirolimus on Naïve and CMV-Specific T Cells Extending Its Applicable Range Beyond Immunosuppression
Source: Front Immunol. 2018 Dec 17;9:2953. doi: 10.3389/fimmu.2018.02953 (PMC6304429; doi:10.3389/fimmu.2018.02953)
Supplement: Supplementary file 1 [file Table_1.docx]

**Table S1.**

**Patient characteristics**

| **No** | **Age** | **Gender** | **Transplantation** | **Donor** | **Immunosuppressive regimen** |
| --- | --- | --- | --- | --- | --- |
| 1 | 12 | Male | stem cell | related haploidentical | Sirolimus |
| 2 | 14 | Male | stem cell | matched sibling | Sirolimus/Prednisolon |
| 3 | 16 | Male | kidney | deceased | Sirolimus/Prednisolon |
| 4 | 16 | Female | kidney | living | Sirolimus/Prednisolon/ Tacrolimus |
| 5 | 16 | Female | kidney | deceased | Sirolimus/Prednisolon |
