# Supplementary material for: Selective Effects of mTOR Inhibitor Sirolimus on Naïve and CMV-Specific T Cells Extending Its Applicable Range Beyond Immunosuppression
Source: Front Immunol. 2018 Dec 17;9:2953. doi: 10.3389/fimmu.2018.02953 (PMC6304429; doi:10.3389/fimmu.2018.02953)
Supplement: Supplementary file 2 [file Data_Sheet_1.docx]

Figure S1.

A

B

C


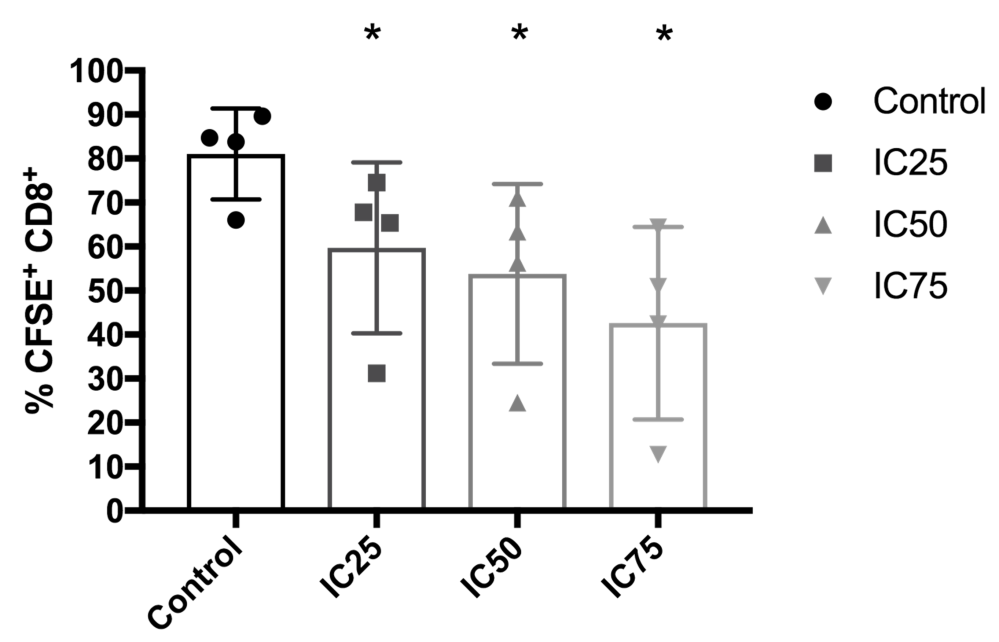

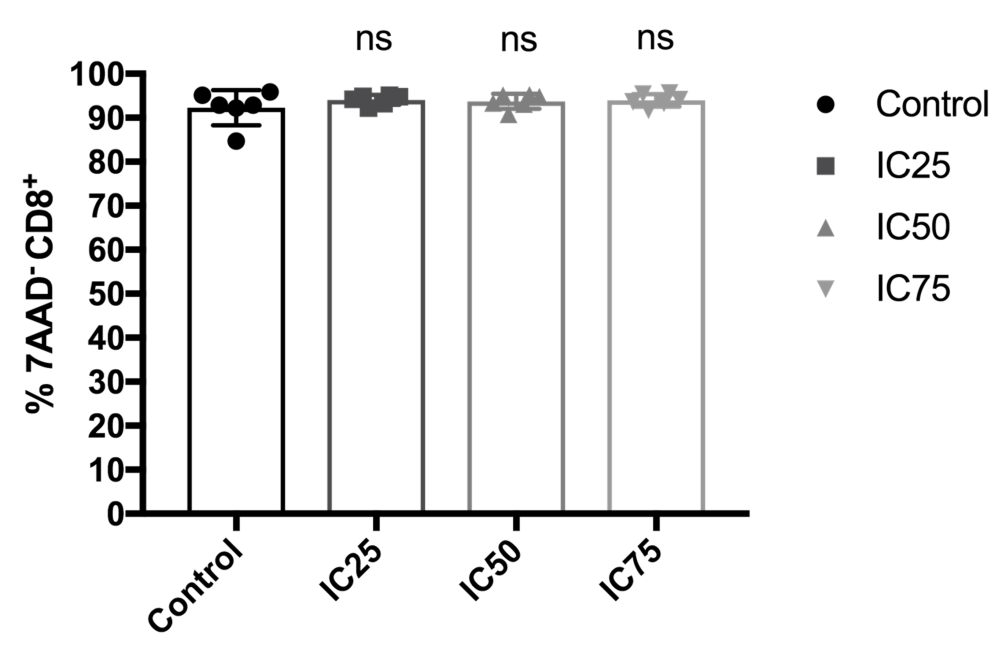

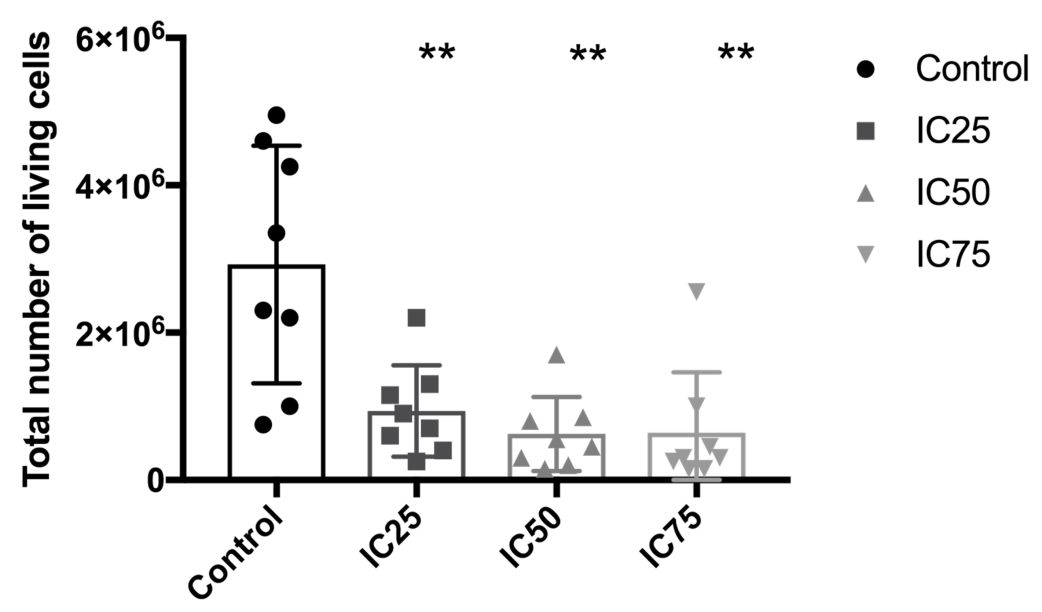


A

B

Figure S2.


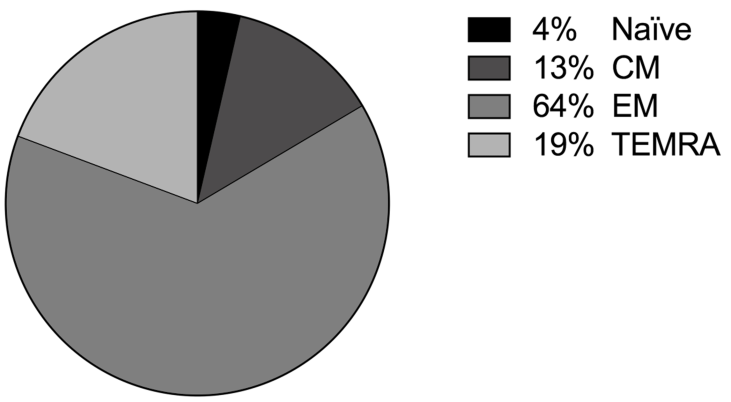

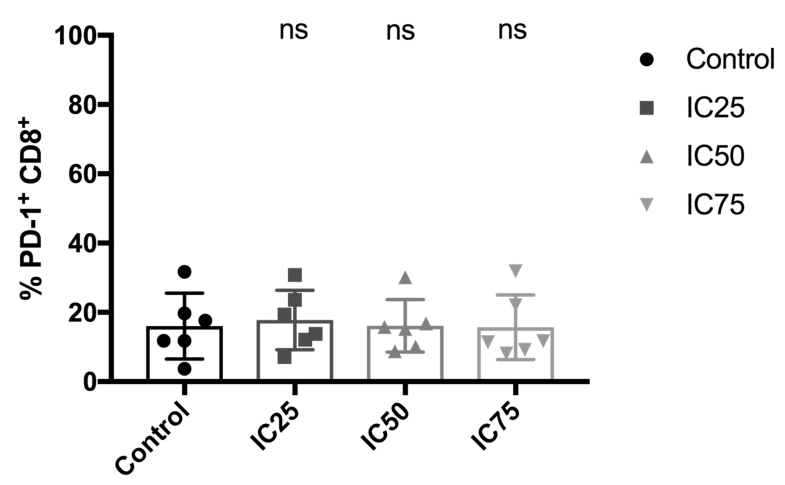

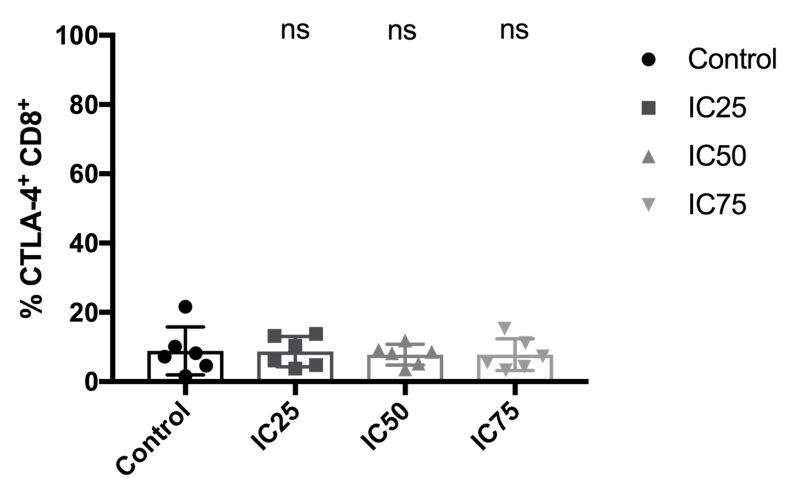

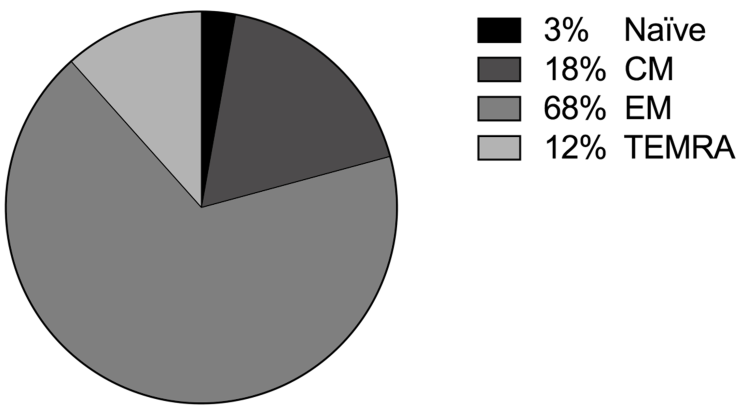

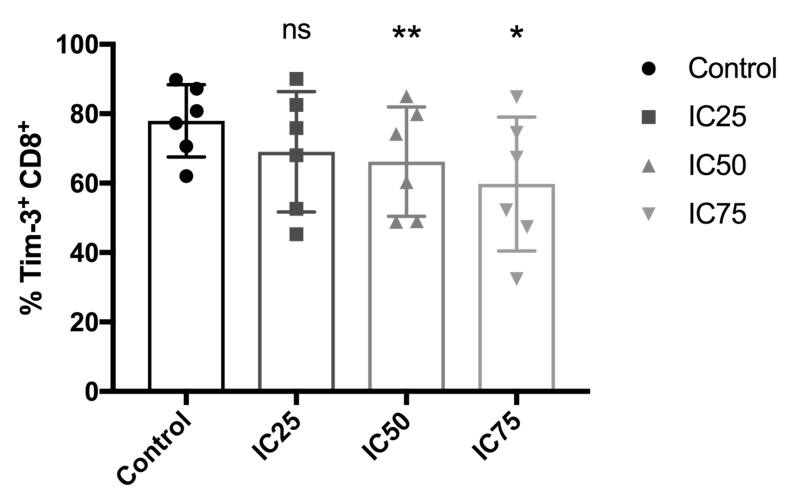

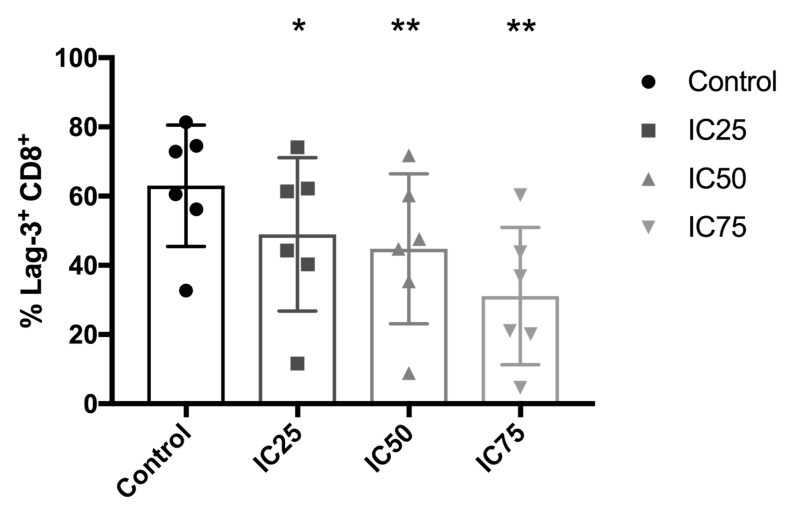

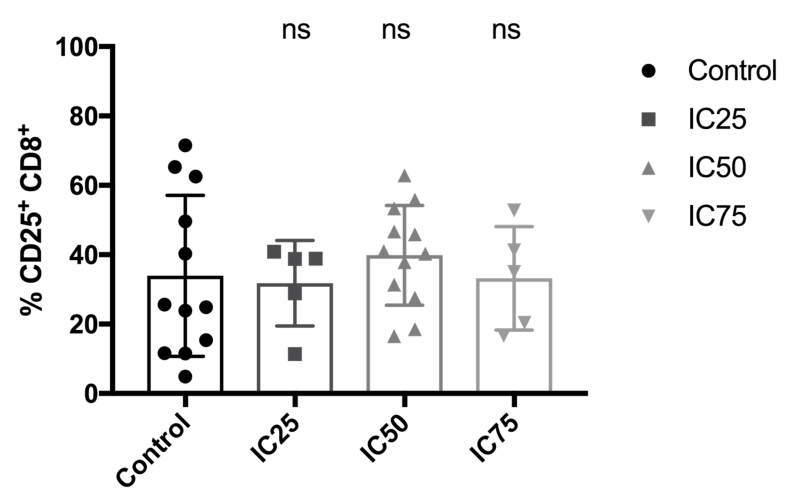

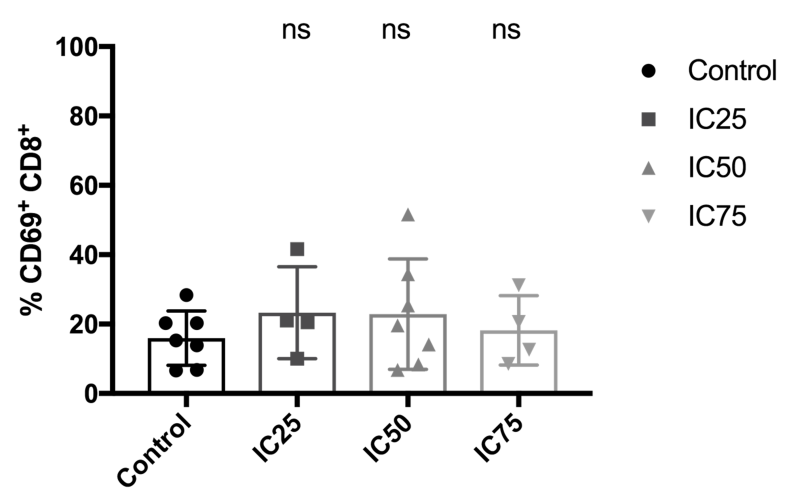


Control

IC50


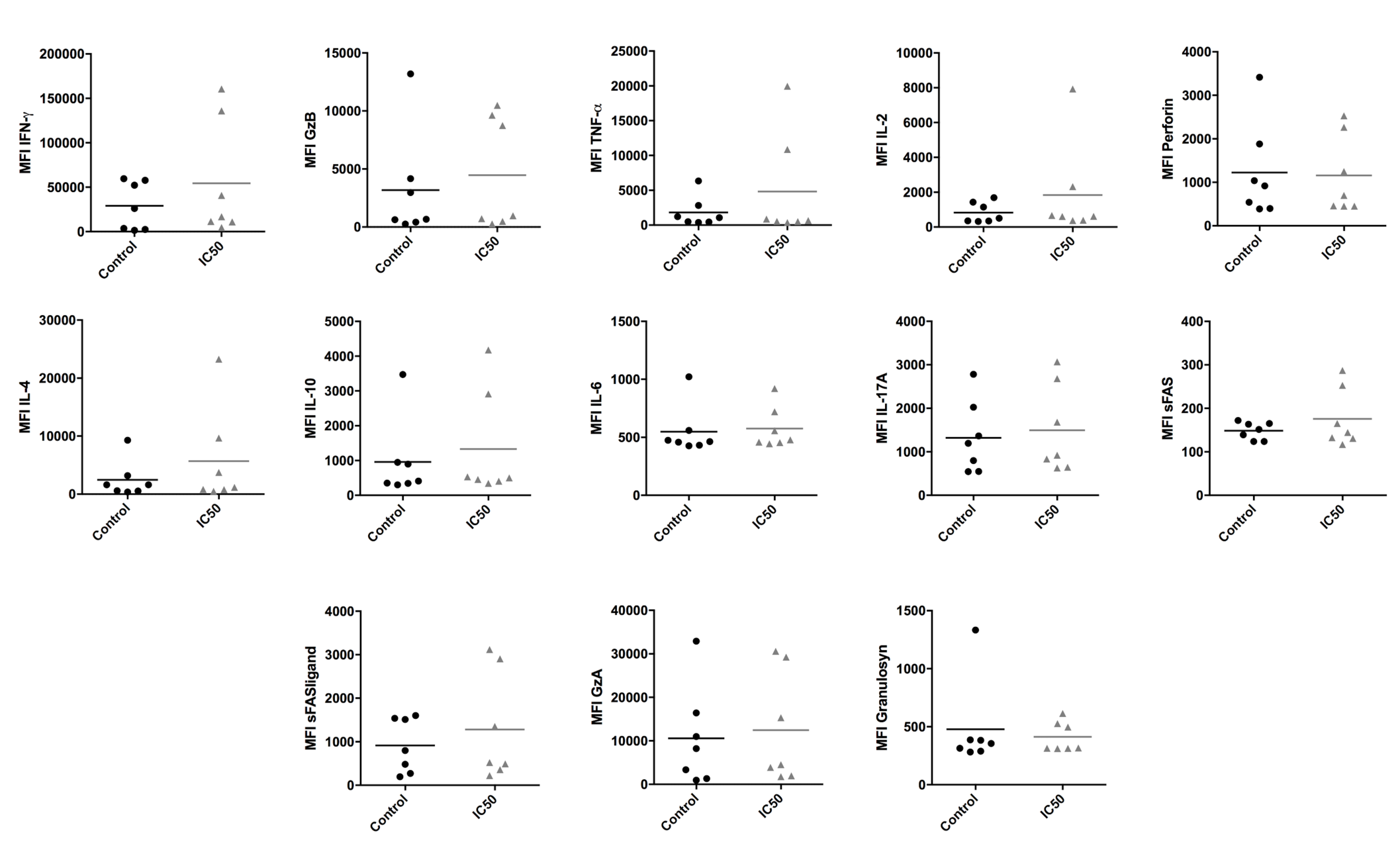


B


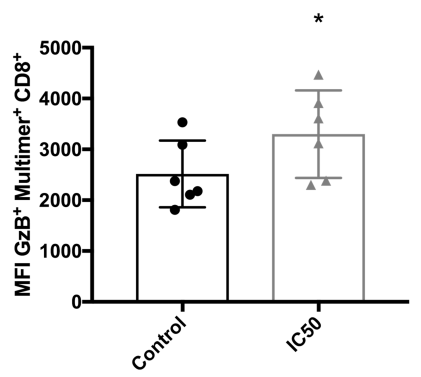

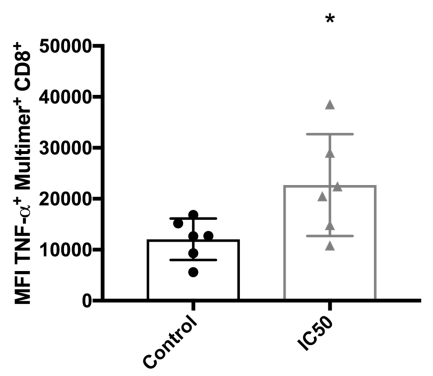

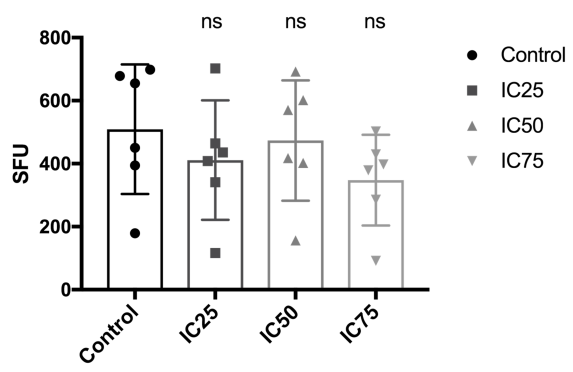

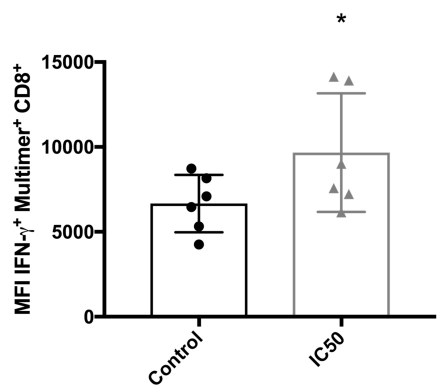


Figure S3.

A

C


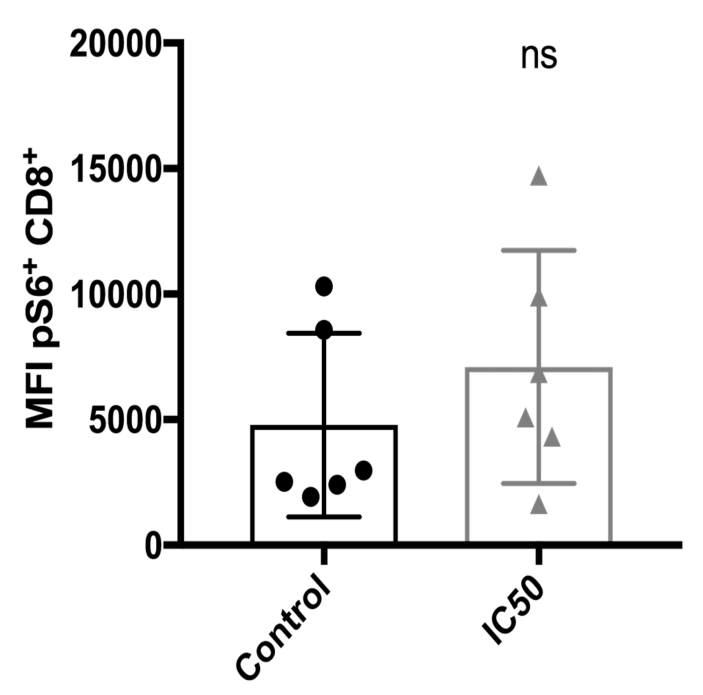

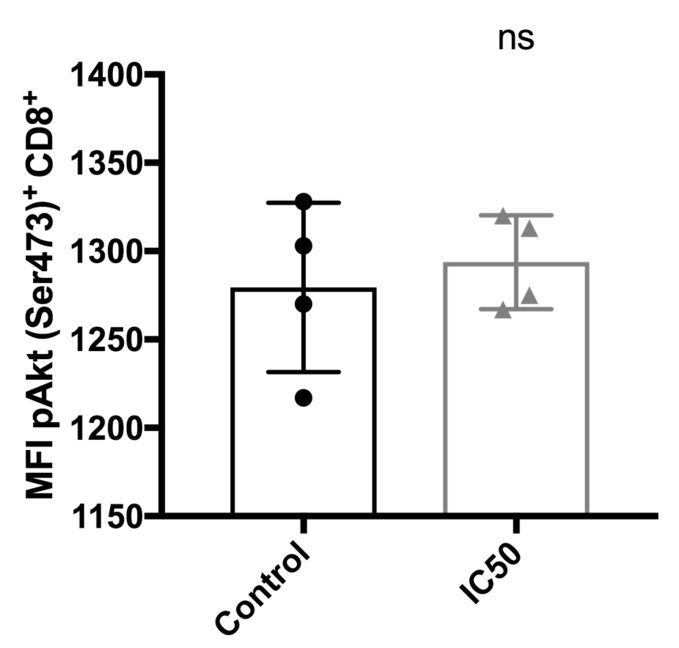

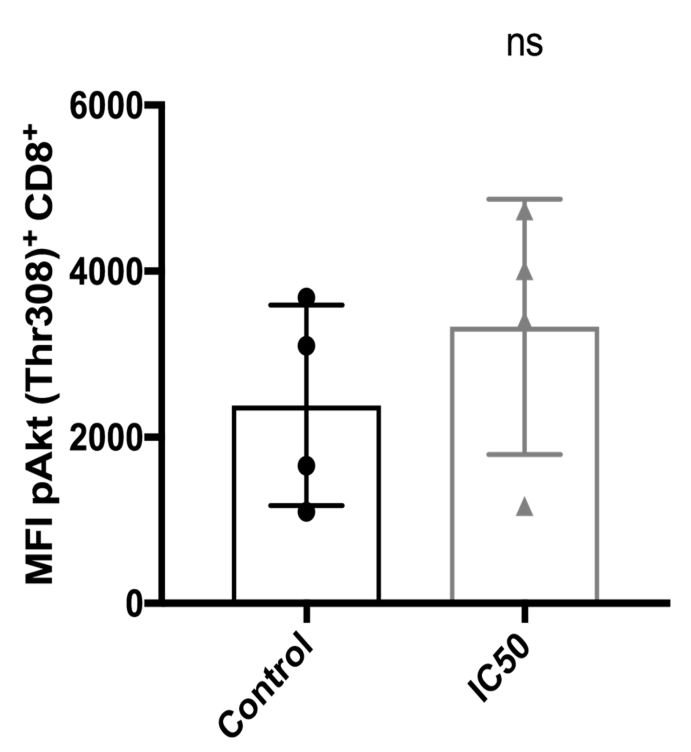

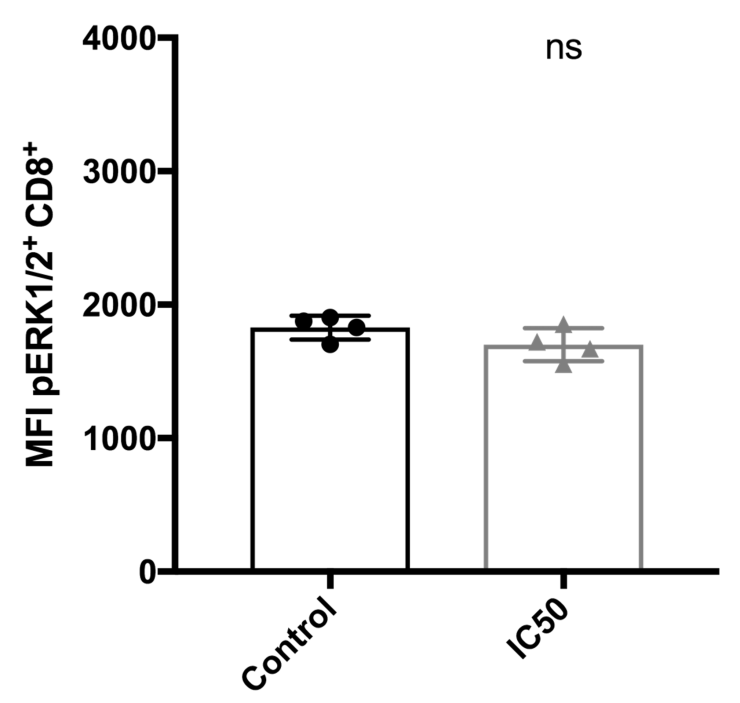

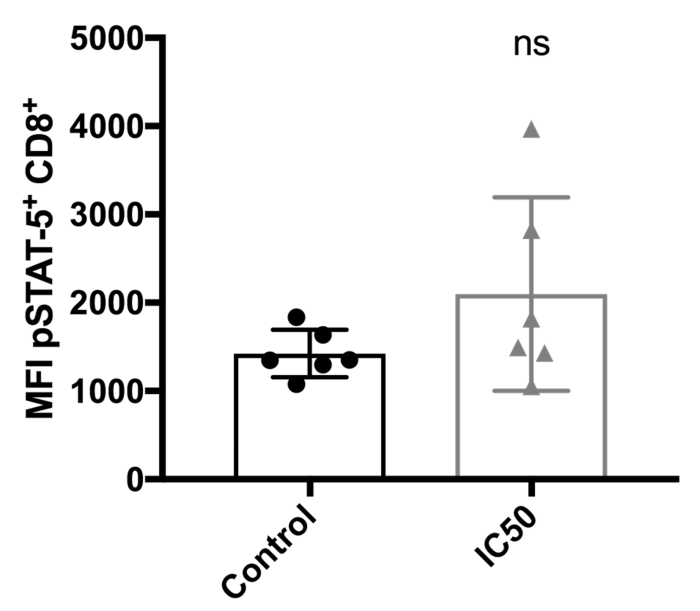


B

Figure S4.

A

C

D

E


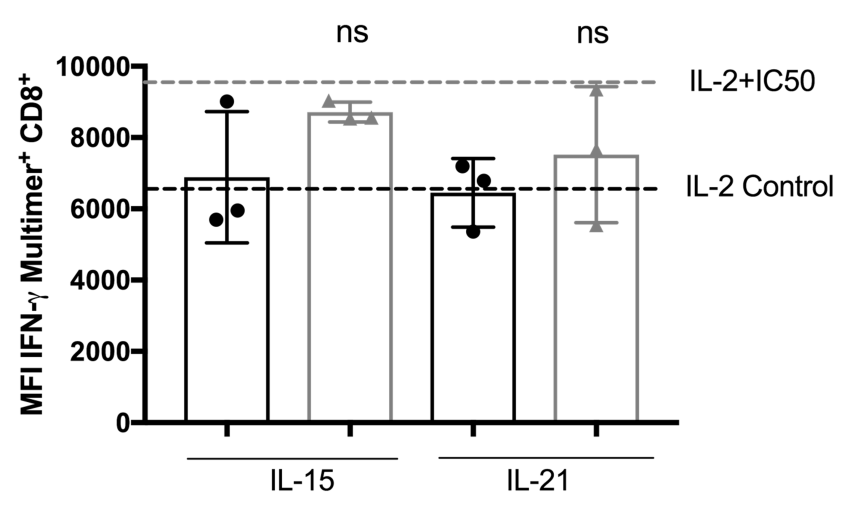

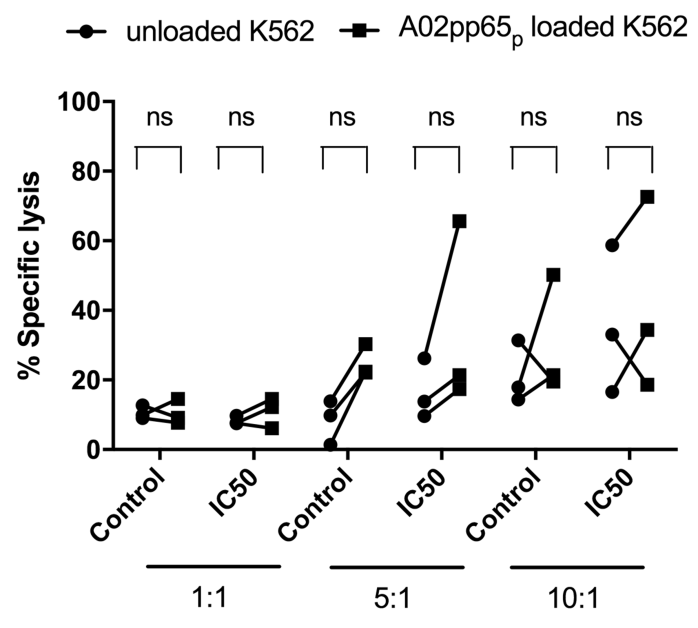

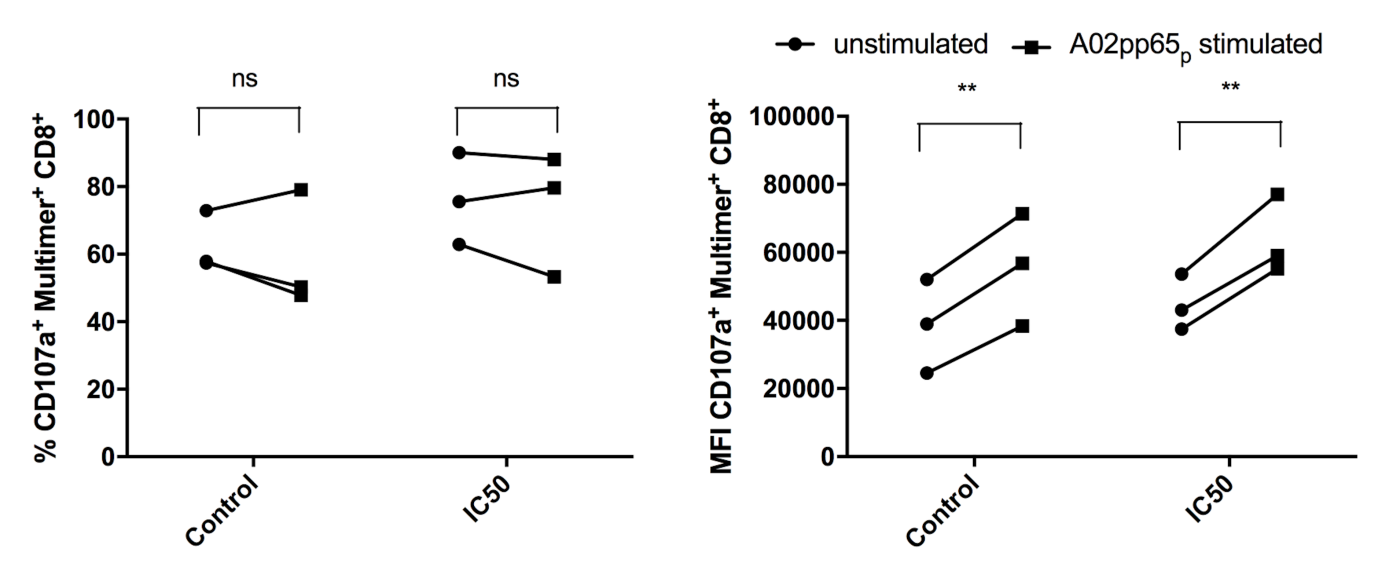

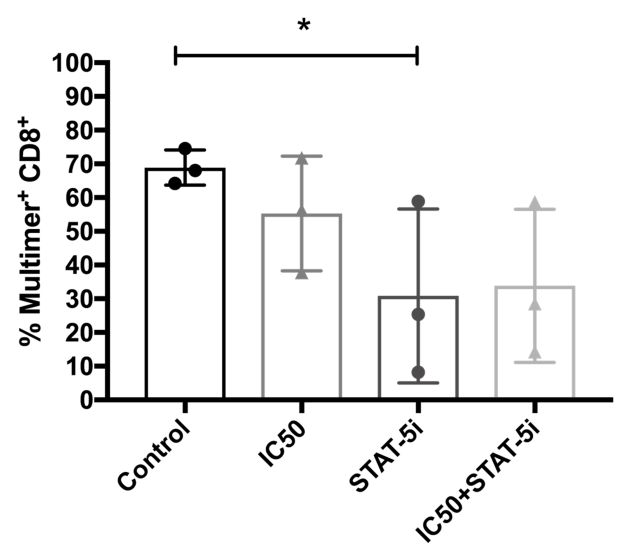

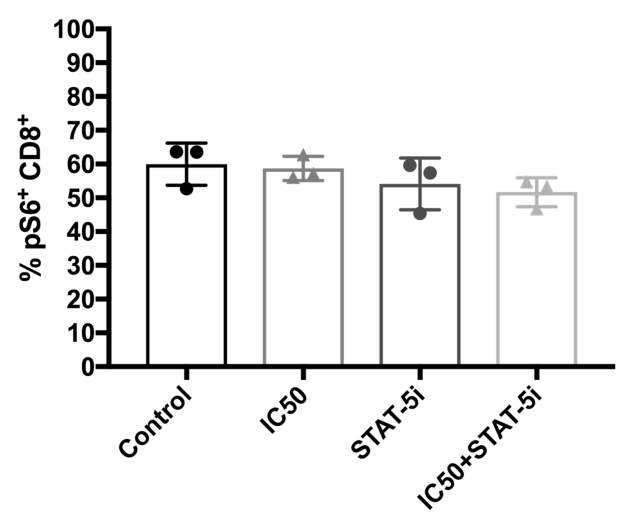

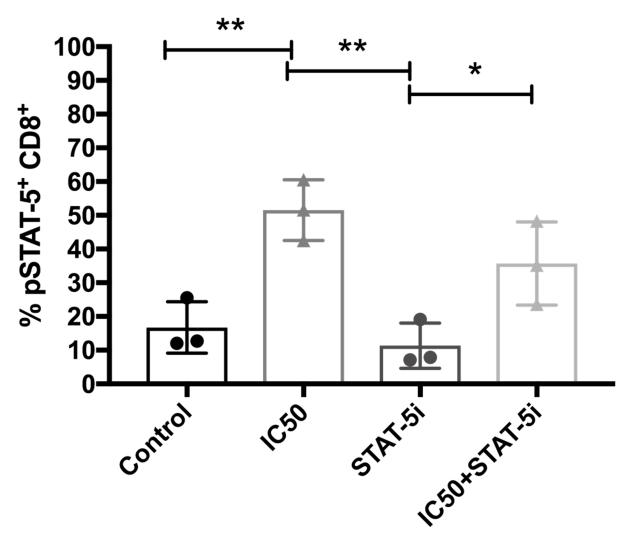

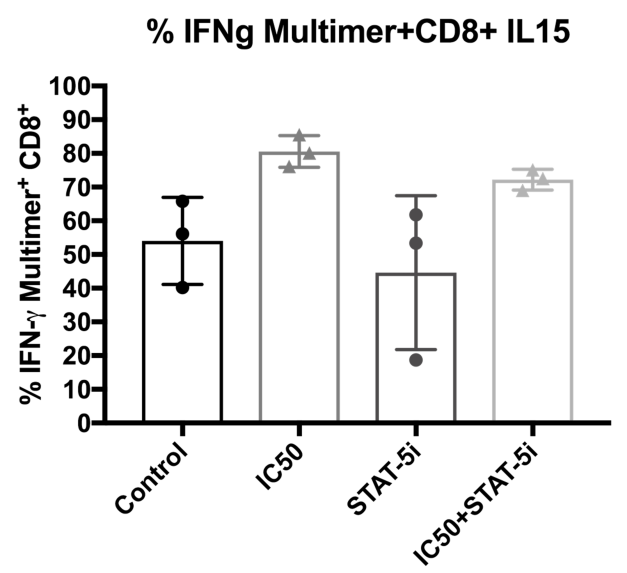


B

Figure S5.

A

C

D

E

F

**Supplementary Figure Legends**

**Figure S1. Negative impact of sirolimus on total CD8^+^ T-cell proliferation.** (**A**) Percentages (%) of proliferated CFSE^+^CD8^+^ T cells were determined by multicolor flow cytometry on day 7 after aAPC stimulation and treatment with sirolimus (IC25, IC50, IC75). Cells treated without sirolimus served as the controls. (**B**) Total numbers of living cells, as determined by trypan blue dye exclusion on day 7. (**C**) Percentages of viable 7AAD^-^CD8^+^ T cells (n=6). Data are shown as means ± SD. The two-paired Student’s t-test was used to test for statistically significant differences [*p<0.05, **p<0.01, non-significant (ns)].

**Figure S2. Sirolimus treatment has no effect on total CD8^+^ T-cell effector memory phenotype.** (**A**) Total CD8^+^ T-cell phenotyping was performed using CD62L and CD46RA surface markers on day 7 after aAPC stimulation without (control) or with IC50 sirolimus treatment (IC50) in the presence of IL-2. CM=central memory T cells; EM=effector memory T cells; TEMRA=effector memory T cells expressing CD45RA, determined as percentages (%) (n=4). (**B**) Percentages (%) of expression of PD-1, CTLA-4, Tim-3, Lag-3, CD25 and CD69 on total CD8^+^ T cells. Data are shown as means ± SD. The two-paired Student’s t-test was used to test for statistically significant differences [**p<0.01, non-significant (ns)].

**Figure S3. Functional improvement on CMV-specific T cells after sirolimus treatment.** (**A**) IFN-γ expression, determined as the number of spot-forming units (SFU) by IFN-γ ELISpot assay. (**B**) Median fluorescence intensity (MFI) of intracellular IFN-γ, GzB and TNF-α on CMV-specific CD8^+^ T cells, as determined by multicolor flow cytometry. (**C**) Median fluorescence intensity of effector cytokines from cell culture supernatant determined by LEGENDplex™ bead-based immunoassay following overnight A02pp65_p_ re-stimulation on 7 days aAPC stimulated and sirolimus treated or untreated CD8^+^ T cells. The data are shown as means ± SD. The two-paired Student’s t-test was used to test for statistically significant differences [*p<0.05, non-significant (ns)].

**Figure S4. Sirolimus treatment alters phosphorylation of signaling molecules.** Median fluorescence intensity (MFI) of phosphorylated (**A**) S6, (**B**) ERK1/2, (**C**) Akt^Thr308^, (**D**) Akt^Ser473^ and (**E**) STAT-5, as determined by phospho-flow cytometry. Values are displayed as mean (±) SD. The two-paired Student’s t-test was used to test for statistically significant differences [*p<0.05, **p<0.01, ***p<0.001, non-significant (ns)].

**Figure S5. CMV-specific T cells became more susceptible to IL-2 or IL2R-sharing cytokines via STAT-5 activation during mTORC1 inhibition.** (**A**) Median fluorescence intensity (MFI) of IFN-γ following 5h of peptide re-stimulation, as determined by intracellular cytokine staining on CMV-specific CD8^+^ T cells after 7 days of aAPC stimulation with or without sirolimus treatment in the presence of IL-2/IL-15/IL-21. Results for supplemented IL-2 without sirolimus (black dashed lines) and with sirolimus (grey dashed lines) are shown (n=4). (**B**) Target cell recognition in IL-15 supplemented cells with or without sirolimus was evaluated on day 7. Total CD8^+^ T cells were co-cultured for 5h with A02pp65_p_-loaded and CFSE labeled A*02-transduced K562 target cells (squares) at effector to target ratios of 1:1, 5:1 and 10:1. Unloaded K562 cells served as controls (spheres). The percentage of cell death was determined by 7AAD staining and multicolor flow cytometric analysis. (**C**) The percentages (%) and MFI of CD107a expression on CMV-specific CD8^+^ T cells was determined by multicolor flow cytometry following 4h of re-stimulation with A02pp65_p_. (**D**) Percentages of expanded CMV-specific CD8^+^ T cells found following overnight STAT-5 inhibition (STAT-5i) on day 7 after aAPCs stimulation with or without sirolimus in the presence of IL-15. (**E**) Percentages of pS6 and pSTAT-5, as determined by phospho-flow cytometry. (**F**) Intracellular expression of IFN-γ (%) following 5h of peptide re-stimulation, as measured by multicolor flow cytometry. Values are displayed as mean (±) SD. Statistical method: (**A**) Student’s t-test and (**B, C, D, E, F**) two-way analysis of variance [*p<0.05, **p<0.01, non-significant (ns)].

A
